# Inflammasome activation and metabolic remodelling in p16‐positive aging cells aggravates high‐fat diet‐induced lung fibrosis by inhibiting NEDD4L‐mediated K48‐polyubiquitin‐dependent degradation of SGK1

**DOI:** 10.1002/ctm2.1308

**Published:** 2023-06-21

**Authors:** Xin Gu, Haoyu Meng, Chengyi Peng, Shiyu Lin, Baihong Li, Lin Zhao, Xue Yang, Guangyan Wang, Wenyuan Cai, Jiawen Zhou, Shuiyuan Liu, Peng Wu, Yingqiang Du, Jianliang Jin, Xiaoyan Wang

**Affiliations:** ^1^ Department of Cardiology Affiliated Hospital of Jiangnan University Wuxi Jiangsu P. R. China; ^2^ Department of Human Anatomy; Research Centre for Bone and Stem Cells; Key Laboratory for Aging & Disease; The State Key Laboratory of Reproductive Medicine Nanjing Medical University Nanjing Jiangsu P. R. China; ^3^ Department of Cardiology, Affiliated Suzhou Hospital of Nanjing Medical University; Suzhou Municipal Hospital; Gusu School Nanjing Medical University Suzhou Jiangsu P. R. China; ^4^ Department of Cardiology First Affiliated Hospital of Nanjing Medicial University Nanjing Jiangsu P. R. China; ^5^ Department of Rheumatology, Nanjing Drum Tower Hospital Affiliated Hospital of Medical School, Nanjing University Nanjing Jiangsu P. R. China; ^6^ Department of Cardiology, Affiliated Nanjing Hospital of Nanjing Medical University Nanjing First Hospital Nanjing Jiangsu P. R. China

**Keywords:** high‐fat diet, inflammaging, NEDD4L, p16, pulmonary fibrosis, SGK1, ubiquitin degradation

## Abstract

**Background:**

Chronic changes caused by a high‐fat diet (HFD) may be associated with weakened lung function in obese patients. However, few studies have focused on the role of senescent cells in HFD‐induced pulmonary fibrosis. This study aimed to determine whether (i) obesity causes the accumulation of aging cells in the lungs, (ii) p16 accumulation in aging epithelial cells or fibroblasts exacerbates long‐term HFD‐induced senescence‐associated pulmonary fibrosis (SAPF) and (iii) *p16* deletion or clearance of aging cells ameliorates HFD‐induced SAPF through inactivation of the inflammasome and metabolic remodelling.

**Methods:**

Twelve‐month old male mice of *p16^INK4a^* (hereafter p16) knockout (*p16^−−^*) and wild‐type (WT), *ApoE* knockout (*ApoE^−−^*) and *ApoE^−−^p16^−−^* were fed a HFD to induce obesity, and the effects of treatment with the senolytic drug ABT263 or the SGK1 specific inhibitor EMD638683 on fibrosis, inflammaging, gene expression, integrin‐inflammasome signalling and metabolism were examined. A549 and IMR‐90 cells were transduced with *p16*‐overexpressing adenovirus, and treated with palmitic and oleic acids (P&O) to induce steatosis in vitro.

**Results:**

We found that long‐term HFD promoted the expression of p16 and the increase of senescent cells in the lung. *P16* knockout or ABT263 treatment alleviated pulmonary fibrosis, the increase of senescent cells and senescence‐associated secretory phenotype (SASP) in HFD‐fed mice, as well as in P&O‐treated A549 and IMR‐90 cells. RNA sequencing and bioinformatics analyses revealed that *p16* knockout inhibited activation of the integrin‐inflammasome pathway and cellular glycolysis. Mass spectrometry, co‐immunoprecipitation and GST pull‐down assays demonstrated that p16 bound to the *N*‐terminal of SGK1, thereby interfering with the interaction between the E3 ubiquitin ligase NEDD4L and SGK1, and subsequently inhibiting K48‐polyubiquitin‐dependent degradation of SGK1 mediated by the NEDD4L–Ubch5 complex. EMD638683 was found to alleviate HFD‐induced pulmonary fibrosis and activation of the integrin‐inflammasome pathway.

**Conclusion:**

P16 accumulation promoted activation of integrin– inflammasome pathway and cell glycolysis by binding to the N– terminal of SGK1, intefering with the interaction between the E3 ubiquitin ligase NEDD4L and SGK1, thereby inhibiting K48– polyubiquitin– dependent degradation of SGK1 mediated by the NEDD4L–Ubch5 complex. ABT263 or EMD638683 could be used as potential drugs to treat pulmonary fibrosis in obese patients.

## Graphical abstract

- Following points are discussed.
- P16 accumulation promoted activation of the integrin‐inflammasome pathway and cell glycolysis by binding to the *N*‐terminal of SGK1.
- P16 interfered with the interaction between NEDD4L and SGK1, thereby inhibiting K48‐polyubiquitin‐dependent degradation of SGK1 mediated by the NEDD4L–Ubch5 complex.
- Clearance of senescent cells by administration of ABT263 or the SGK1‐specific inhibitor EMD638683 or *SGK1* siRNA ameliorated HFD‐induced pulmonary fibrosis.

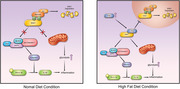

## 1 BACKGROUND

Chronic changes caused by a high‐fat diet (HFD) may be associated with weakened lung functions in obese patients.^1^ Several studies have demonstrated that excessive intake of saturated fatty acids and meat increase incidence risk of idiopathic pulmonary fibrosis (IPF) and other respiratory diseases, and decrease pulmonary functions, suggesting linkage between obesity and IPF.^2^, ^3^, ^4^, ^5^ The mechanisms involved in the initiation and progression of HFD‐induced lung fibrosis are poorly defined and are most likely multi‐factorial. Accumulation of senescent cells in the lungs of IPF and chronic obstructive pulmonary disorder (COPD) patients has been explored in recent years.^6^, ^7^ However, few studies have focused on the role of senescent cells in HFD‐induced pulmonary fibrosis.

Alveolar epithelial type II cells, fibroblasts and endothelial cells have been shown to undergo senescence in IPF,^8^ as well as in various pulmonary fibrosis mouse models.^8^, ^9^ The accumulation of senescent cells aggravates pulmonary fibrosis due to an aggravation in senescence‐associated secretory phenotype (SASP) and oxidative stress. Treatment with senolytic drugs or gene therapy to eliminate senescent cells has been shown to alleviate pulmonary fibrosis.^6^, ^10^ ABT‐263 is a potential senolytic drug that possesses anti‐aging and anti‐profibrotic properties.^11^ Indeed, bleomycin‐induced pulmonary fibrosis was previously found to be ameliorated by ABT‐263 treatment through inhibition of cellular senescence and attenuation of the expression of mitochondrial anti‐viral signalling proteins and their signalling pathways.^12^ Mouse models of progressive fibrosing interstitial lung diseases are also alleviated by ABT‐263 treatment through the induction of fibroblast apoptosis, decreased number of fibroblasts and a reduction in lung collagen levels.^11^ Thus, senescent cells and cellular senescence‐related signalling pathways could be new targets for the treatment of pulmonary fibrosis.

Several studies have found that HFD sensitizes mice to bleomycin‐induced pulmonary fibrosis.^13^, ^14^ In addition, HFD exacerbates bleomycin‐induced pulmonary fibrosis in wild‐type (WT) and *ApoE* knockout (*ApoE^−/−^*) mice.^15^, ^16^ Previous studies have also reported that HFD induces peribronchial and perivascular collagen deposition via activation of the transforming growth factor‐beta‐1 (TGF‐β1) signalling pathway, thereby inducing pulmonary fibrosis regardless of whether mice were exposed to allergens.^15^, ^17^ Long‐term HFD causes pulmonary fibrosis and airway hyper‐responsiveness.^15^, ^18^ Furthermore, pulmonary epithelial‐mesenchymal transition and low‐grade inflammation caused by HFD may be critical manifestations that lead to pulmonary fibrosis.^15^ To date, the mechanism of HFD‐induced pulmonary fibrosis has not been clearly defined. Furthermore, it remains unclear whether (i) HFD causes accumulation of senescent cells in the lung, (ii) accumulation of aging cells occurs in pulmonary fibrosis induced by long‐term HFD, and (iii) removing senescent cells reverses this pathological process.

P16 is a well‐characterized marker of cell senescence, and is significantly activated in IPF and COPD patients.^6^, ^19^ Despite the heterogeneity of senescent cells in multiple organs, p16‐positive senescent cells are still used as an important indicator of senescence, and play a crucial role in the pro‐inflammatory microenvironment and age‐related diseases.^20^, ^21^, ^22^ We previously found that *p16* knockout (*p16^−−^*) rescues senescence‐associated pulmonary fibrosis (SAPF) in a premature senescence mouse model through down‐regulation of the TGF‐β1/IL‐11/MEK/ERK pathway.^23^ In addition to the well‐established cell cycle arrest effect of p16, we recently reported that p16 could bind to ERK1/2 or occludin, thereby interfering with their sub‐cellular translocation and function.^23^, ^24^ However, the role of p16 in regulating HFD‐induced SAPF is still unknown. Our previous study demonstrated that excessive activation of the inflammasome accelerated inflammaging, as shown by an increase in damage‐related molecular patterns in senescent cells.^25^ However, it remains unclear whether *p16* deletion or clearance of senescent cells could ameliorate HFD‐induced SAPF by preventing inflammasome activation.

Metabolic remodelling of senescent cells is critical for the transformation of healthy cells into senescent cells, and is also one of the key reasons that senescent cells promote a pro‐inflammatory microenvironment.^26^ Enhanced cellular glycolysis, a key change in cell metabolism after aging, has been observed in IPF samples.^27^, ^28^ However, it is unclear whether p16 induces metabolic remodelling of senescent cells, and whether *p16* deletion or clearance of aging cells could ameliorate HFD‐induced SAPF through regulation of cell glycolysis.

Our current study found that p16 accumulation promoted activation of the integrin‐inflammasome pathway and cell glycolysis by binding to the *N*‐terminal of SGK1, interfering with the interaction between the E3 ubiquitin ligase NEDD4L and SGK1, thereby inhibiting K48‐polyubiquitin‐dependent degradation of SGK1 mediated by the NEDD4L–Ubch5 complex. Furthermore, based on the specific regulatory signalling pathways identified here, we demonstrated that clearance of senescent cells by administration of ABT263 or the SGK1‐specific inhibitor EMD638683 ameliorated HFD‐induced pulmonary fibrosis. Thus, ABT263 and EMD638683 could be used as potential drugs to treat pulmonary fibrosis in obese patients.

## 2 MATERIALS AND METHODS

All materials and methods are described in detail in Supporting Information.

## 3 RESULTS

### 3.1 HFD induces p16 accumulation and cell senescence of epithelial cells and fibroblasts in the lungs of aging WT or *ApoE* ^−/−^ mice

**FIGURE 1:**
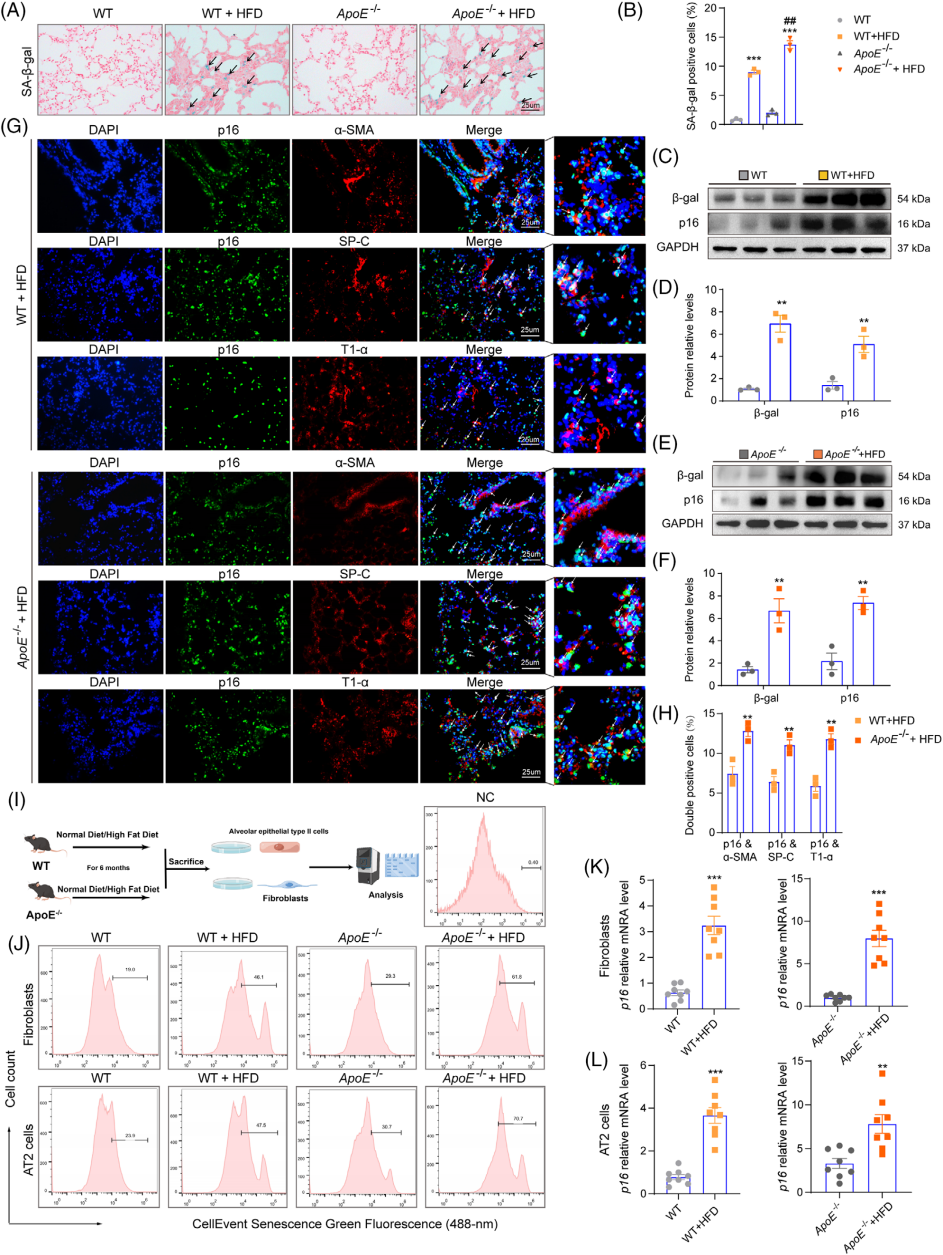
HFD induces p16 accumulation and cell senescence in epithelial cells and fibroblasts in the lungs of aging WT or *ApoE^−/−^* mice. Twelve‐month‐old WT and *ApoE^−/−^* mice were fed a HFD for 6 months and lung tissues were collected for further analysis. (A) Representative images showing senescence‐associated‐β‐galactosidase (SA‐β‐gal) staining in the lung tissue of different treatment groups. Nuclei were visualised with Nuclear Fast Red staining. (B) Percentage of cells positive for SA‐β‐gal. n = 3 mice per group. Values are given as mean ± SEM, ***p < .001 compared with WT or *ApoE^−/−^* mice; ^#^ *p* < .05 compared with WT mice on HFD. Statistical analysis was performed using one‐way ANOVA. (C) Representative western blot showing β‐gal and p16 protein expression levels in the lung tissue of different treatment groups. GAPDH was used as the loading control. (D) Protein bands were quantified by densitometric analysis and normalized to GAPDH levels. (E) Representative western blot showing β‐gal and p16 protein expression levels in the lung tissue of different treatment groups. GAPDH was used as the loading control. (F) Protein bands were quantified by densitometric analysis and normalized to GAPDH levels. n = 3 biological replicates per experiment. Values are given as mean ± SEM, **p < .01 compared with WT or *ApoE^−/−^* mice. Statistical analysis was performed using Student's *t*‐test. (G) Representative immunofluorescence images showing p16 protein expression in pulmonary fibroblasts (α‐SMA^+^), alveolar type II epithelial cells (SP‐C^+^), and alveolar type I epithelial cells (T1‐α^+^). (H) The percentages of p16 & α‐SMA, p16 & SP‐C and p16 & T1‐α double positive cells. n = 3 mice per group. Values are given as mean ± SEM, **p < .001 compared with WT mice on HFD. Statistical analysis was performed using Student's *t*‐test. (I) Primary fibroblasts and type II alveolar epithelial cells were obtained from 12‐month‐old WT and *ApoE^−/−^* mice on HFD or normal diet for 6 months. (J) Flow cytometry analysis of SA‐β‐gal activity was performed in primary fibroblasts and type II alveolar epithelial cells using the CellEvent™ Senescence Green Probe and 488‐nm laser. (K–L) RT‐qPCR analysis of *P16* mRNA levels in primary fibroblasts and type II alveolar epithelial cells. Values were calculated relative to the *GAPDH* mRNA loading control. n = 8 biological replicates per experiment. Values are given as mean ± SEM, **p < .01, ***p < .001 compared with WT or *ApoE^−/−^* mice. Statistical analysis was performed using Student's *t*‐test.

To determine whether HFD induces cell senescence in the lung, physiological aging was induced in 12‐month‐old WT and *ApoE^−−^* mice by feeding them a HFD for 6 months. We found a significant increase of senescence‐associated‐β‐galactosidase (SA‐β‐gal)‐positive cells, as well as β‐gal and p16 protein levels in HFD‐fed WT (or *ApoE^−−^*) mice compared with WT (or *ApoE^−−^*) mice (Figure 1A–F). To identify the type of senescent cells in the lungs of HFD mice, lungs were stained with antibodies against α‐SMA, T1‐α or SP‐C as markers for lung fibroblasts, type I or II alveolar epithelial cells, respectively, and co‐localized with p16. We found that HFD induced cell senescence of pulmonary fibroblasts, as well as type I and II alveolar epithelial cells and the percentages of p16 & α‐SMA, p16 & SP‐C and p16 & T1‐α double positive cells elevated in *ApoE^−−^* mice fed a HFD compared to WT mice fed with a HFD (Figure 1G and H). Next, we isolated and cultured type II alveolar epithelial cells and pulmonary fibroblasts from 12‐month‐old *ApoE^−−^* and WT mice fed a HFD for 6 months (Figure 1I). Significantly higher SA‐β‐gal activity and *p16*, *p19*, *p21* and *p53* mRNA expression levels were found in the lung fibroblasts and type II alveolar epithelial cells derived from HFD mice than mice fed a normal diet in both WT and *ApoE^−−^* cells (Figure 1J–L and Supporting Information Figure S1A and B). Next, we treated human embryonic lung fibroblasts (IMR‐90) and human alveolar epithelial cells (A549) with sodium palmitate and sodium oleate (P&O) to induce an in vitro model of non‐inflammatory steatosis. We found that induction of steatosis led to increased p16 and β‐gal protein expression levels in A549 and IMR‐90 cells (Supporting Information Figure S1C–F). Taken together, our findings demonstrated that p16 accumulation and cell senescence of epithelial cells and fibroblasts in the lung were induced by feeding mice a HFD in vivo and by treating IMR‐90 and A549 cells with P&O in vitro.

### 3.2 *P16* deletion or ABT263 treatment inhibits HFD‐induced pulmonary fibrosis in aging WT or *ApoE^−/−^* mice

**FIGURE 2:**
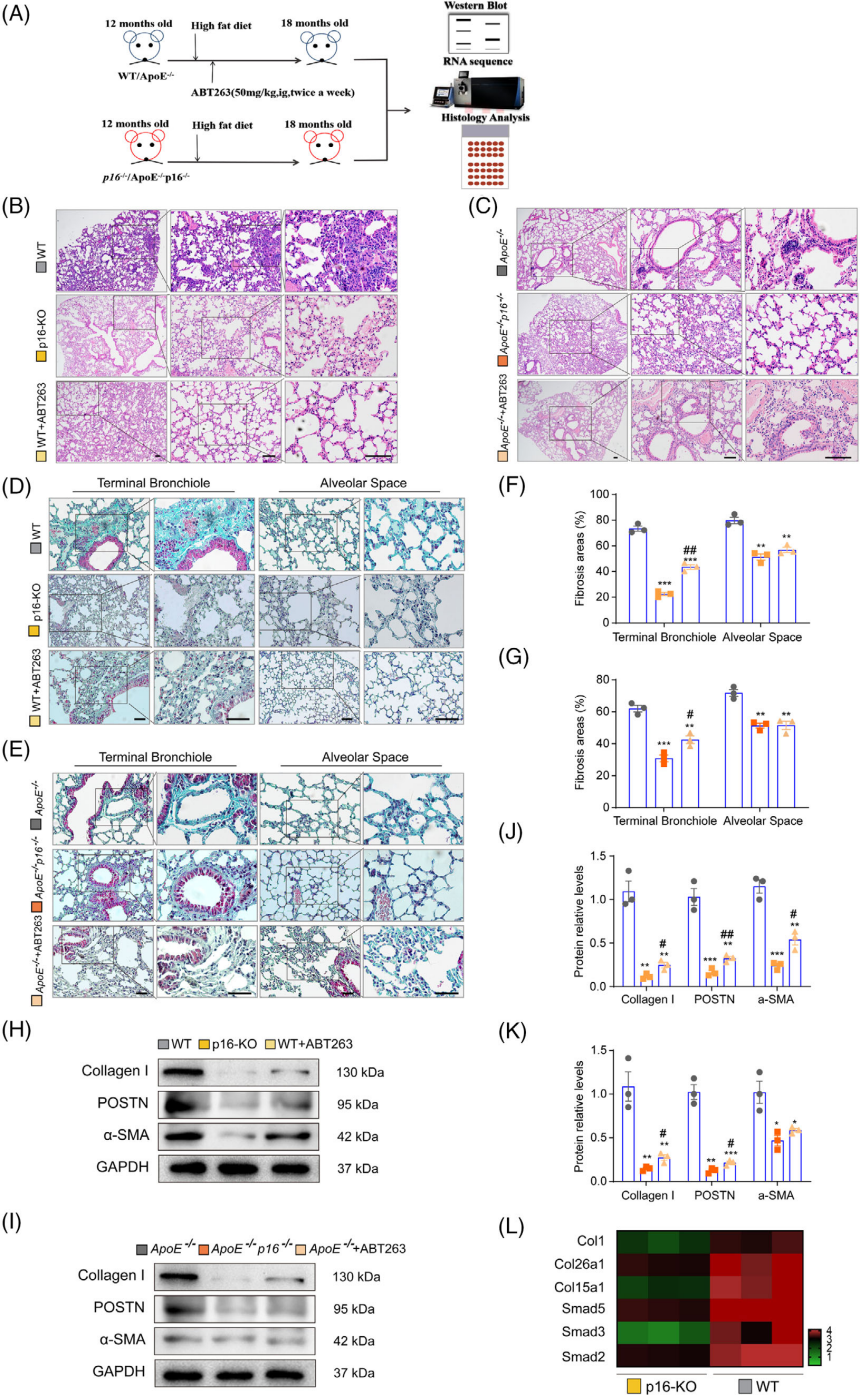
*P16* deletion or ABT263 treatment inhibits HFD‐induced pulmonary fibrosis in aging WT or *ApoE^−/−^* mice. Twelve‐month‐old WT and *ApoE^−/−^* mice were fed a HFD and treated with ABT263 for 6 months. Twelve‐month‐old *p16^−/−^* and *ApoE^−/−^p16^−/−^* mice were fed a HFD without ABT263 treatment. (A) Lung tissues obtained from these mice were subsequently analysed with histological, molecular biology and transcriptome sequencing assays. (B–E) Representative images showing lung tissue sections stained with H&E (B,C) and Masson's trichrome (Masson) stain (D,E). (F,G) Percentage of terminal bronchial and alveolar areas stained positive for Masson's trichrome stain in D and E, respectively. (H,I) Representative western blot showing collagen I, periostinin (POSTN) and α‐SMA protein expression levels in the lung tissue of different treatment groups. GAPDH was used as the loading control. (J,K) Protein bands were quantified by densitometric analysis and normalized to GAPDH levels. n = 3 mice per group. Values are given as the mean ± SEM, *p < .05, **p < .01, ***p < .001 compared with WT or *ApoE^−/−^* mice; ^#^ *p* < .05, ^##^ *p* < .01 compared with *p16^−/−^* or *ApoE^−/−^p16^−/−^* mice. Statistical analysis was performed using one‐way ANOVA. (L) Heatmap showing the differentially expressed genes identified by RNA‐seq analysis including the pro‐fibrotic genes (*Col1*, *Col26a1*, *Col15a1*), and downstream targets of the TGF‐β1 signalling pathway (*Smad5*, *Smad3* and *Smad2*).

To confirm that p16 has a role in HFD‐induced lung fibrosis, 12‐month‐old WT and *ApoE^−/−^* mice were fed a HFD diet and administered with ABT263 for 6 months, while 12‐month‐old *p16^−/−^* and *ApoE^−/−^p16^−/−^* mice were fed a HFD without ABT263 treatment. Lung tissue samples from the 18‐month‐old mice were then examined using histological, molecular biology and transcriptome sequencing assays (Figure 2A). H&E and Masson's trichrome staining revealed that pulmonary inflammatory cell infiltration and fibrosis were obviously decreased in HFD‐fed *p16^−/−^* (or *ApoE^−/−^p16^−/−^*) mice and HFD+ABT263‐treated WT (or *ApoE^−/−^*) mice compared to HFD‐fed WT (or *ApoE^−/−^*) mice (Figures 2B–G and Supporting Information Figure S2A–D). Furthermore, a significant reduction in collagen I, periostin (POSTN) and α‐SMA protein expression levels (Figure 2H–K), and POSTN‐ and α‐SMA‐positive areas of the lung (Supporting Information Figure S3A–F) were observed in HFD‐fed *p16^−/−^* (or *ApoE^−/−^p16^−/−^*) mice and HFD+ABT263‐treated WT (or *ApoE^−/−^*) mice compared to HFD‐fed WT (or *ApoE^−/−^*) mice. Next, lung tissues from HFD‐fed 12‐month‐old WT and *p16^−/−^* mice were analysed by RNA‐seq. We found that *p16* deletion down‐regulated mRNA of *Col1*, *Col26a1*, and *Col15a1*, as well as the downstream targets of TGF‐β1 such as *Smad2*, *Smad3* and *Smad5* (Figure 2L). Our results indicated that *p16* knockout resulted in protective effects against pulmonary fibrotic events in HFD‐fed mice. In addition, ABT263 eliminated senescent cells to alleviate HFD‐induced pulmonary fibrosis.

The pro‐fibrotic effects of p16 during non‐inflammatory steatosis were examined in vitro in *p16*‐overexpressing IMR‐90 and A549 cells that had been treated with P&O. A significant increase in α‐SMA‐ and POSTN‐positive staining, and α‐SMA protein levels was observed in the *p16* over‐expression group compared to the vehicle group (Supporting Information Figure S3G–N), suggesting that p16 may have a pro‐fibrotic effect on pulmonary cells.

### 3.3 *P16* deletion or ABT263 treatment ameliorates cell senescence and SASP in the lungs of mice fed a HFD

**FIGURE 3:**
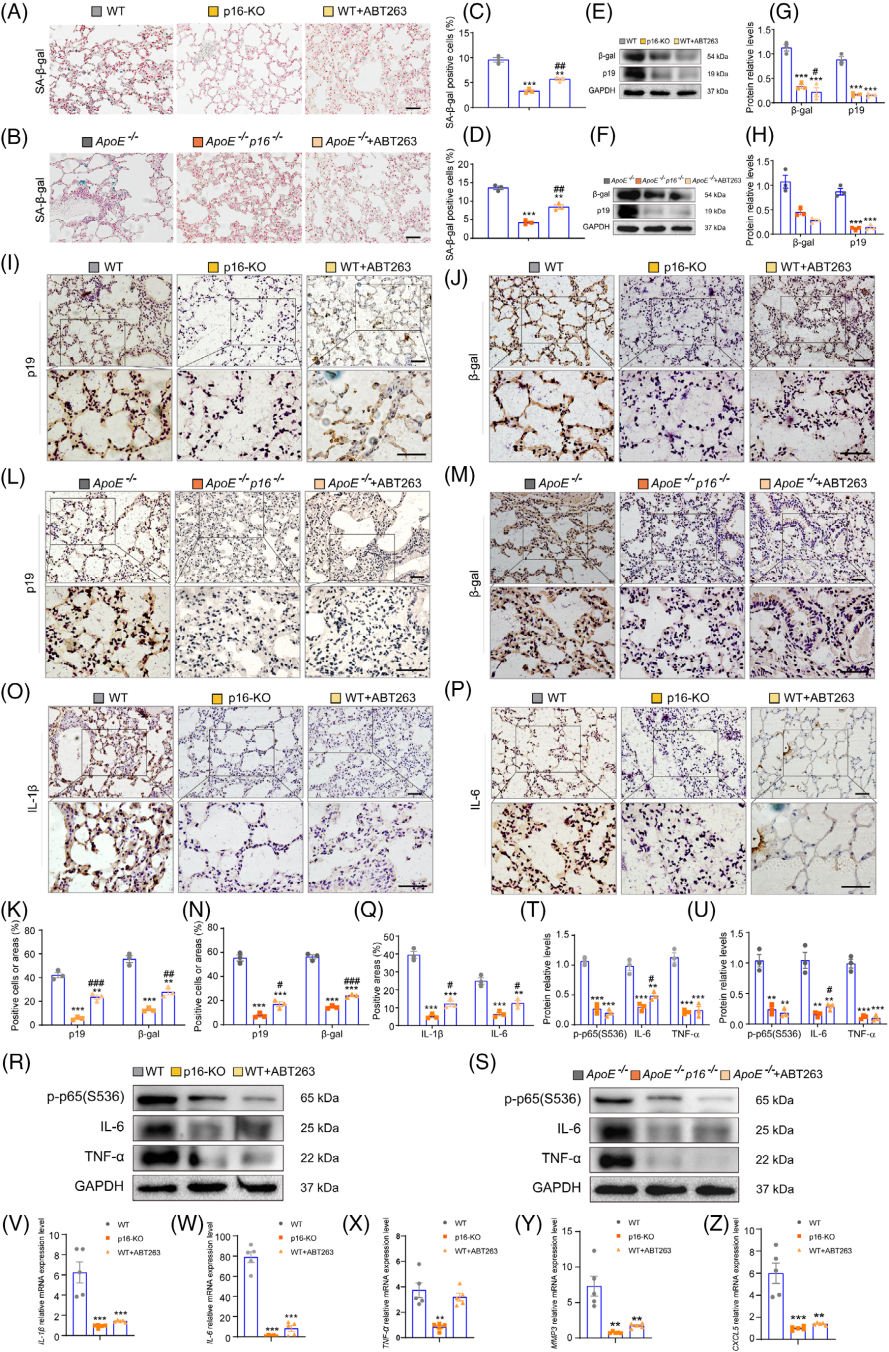
*P16* deletion or ABT263 treatment ameliorates cell senescence and SASP in the lungs of HFD‐fed mice. Twelve‐month‐old WT and *ApoE^−/−^* mice were fed a HFD and treated with ABT263 for 6 months. Twelve‐month‐old *p16^−/−^* and *ApoE^−/−^p16^−/−^* mice were fed a HFD without ABT263 treatment. (A,B) Representative images showing SA‐β‐gal staining in the lung tissue of different treatment groups. Nuclei were visualised with Nuclear Fast Red staining. (C,D) Percentage of cells positive for SA‐β‐gal in A and B, respectively. n = 3 mice per group. Values are given as mean ± SEM, **p < .01, ***p < .001 compared with WT or *ApoE^−/−^* mice; ^##^ *p* < .01, ^###^ *p* < .001 compared with *p16^−/−^* or *ApoE^−/−^p16^−/−^* mice. Statistical analysis was performed using one‐way ANOVA. (E,F) Representative western blot showing β‐gal and p19 protein expression levels in the lung tissue of different treatment groups. GAPDH was used as the loading control. (G,H) Protein bands were quantified by densitometric analysis and normalized to GAPDH levels. n = 3 biological replicates per experiment. Values are given as the mean ± SEM, ***p < .001 compared with WT or *ApoE^−/−^* mice; ^#^ *p* < .05 compared with *p16^−/−^* mice. Statistical analysis was performed using one‐way ANOVA. (I,J) Representative immunohistochemical images showing p19 and β‐gal staining in the lungs of 18‐month‐old *p16^−/−^*, WT and WT+ABT263 mice. (K) Percentage of cells or areas positive for p19 and β‐gal in I and J, respectively. (L–M) Representative immunohistochemical images showing p19 and β‐gal staining in the lungs of 18‐month‐old *ApoE^−/−^*, *ApoE^−/−^p16^−/−^* and *ApoE^−/−^* +ABT263 mice. (N) Percentage of cells or areas positive for p19 and β‐gal in L and M, respectively. (O,P) Representative immunohistochemical images showing IL‐1β and IL‐6 staining in the lungs of 18‐month‐old *p16^−/−^*, WT and WT+ABT263 mice. (Q) Percentage of cells or areas positive for p19 and β‐gal in O and P, respectively. (R,S) Representative western blots showing p‐p65(S536), IL‐6 and TNF‐α protein expression levels. GAPDH was used as the loading control. (T,U) Protein bands were quantified by densitometric analysis and normalized to GAPDH levels. n = 3 mice per group. Values are given as mean ± SEM, **p < .01, ***p < .001 compared with WT or *ApoE^−/−^* mice; ^#^ *p* < .05, ^##^ *p* < .01, ^###^ *p* < .001 compared with *p16^−/−^* or *ApoE^−/−^p16^−/−^* mice. Statistical analysis was performed using one‐way ANOVA. (V‐Z) RT‐qPCR was used to assess the mRNA expression levels of SASP genes (*IL‐1β*, *IL‐6*, *TNF‐α*, *MMP3* and *CXCL5*) in the lung tissue of different treatment groups. Values were calculated relative to the *GAPDH* mRNA loading control. n = 5 mice per group. Values are given as mean ± SEM, **p < .01, ***p < .001 compared with WT mice. Statistical analysis was performed using one‐way ANOVA.

Next, we sought to determine whether *p16* deletion or ABT263 treatment could ameliorate cell senescence and SASP in the lungs of aging mice fed a HFD. We found a decrease of SA‐β‐gal‐positive cells (Figure 3A–D), β‐gal and p19 protein expression levels (Figure 3E–H), β‐gal‐ and p19‐positive cells (Figure 3I–N), and *CDK1*, *CCNB2*, *CDKN1C*, *CCNB1*, *p53* and *p21* mRNA levels (Supporting Information Figure S4A–L) in HFD‐fed *p16^−−^* (or *ApoE^−−^p16^−−^*) mice and HFD+ABT263‐treated WT (or *ApoE^−−^*) mice compared with WT (or *ApoE^−−^*) mice fed a HFD. In the in vitro model of non‐inflammatory steatosis, β‐gal or p53 protein levels were significantly increased in the *p16*‐overexpressing IMR‐90 and A549 cells compared to the controls (Supporting Information Figure S5A–D).

Senescent cells display SASP and cause chronic low‐grade inflammation, known as inflammaging. Activation of the NF‐κB pathway reportedly induces secretion of SASP.^29^ Thus, we next examined the effects of aging, HFD and ABT263 treatment on the relationship between p16 and SASP in WT, *ApoE^−/−^*, *p16^−/−^* and *ApoE^−/−^p16^−/−^* mice. Immunohistochemical analysis revealed a significant reduction in IL‐1β‐, IL‐6‐ or TNF‐α‐positive cells from *p16^−/−^* (or *ApoE^−/−^p16^−/−^*) mice fed a HFD and HFD+ABT263‐treated WT (or *ApoE^−/−^*) mice compared with WT (or *ApoE^−/−^*) mice fed a HFD (Figure 3O–Q). In addition, p‐p65(Ser536), IL‐6 and TNF‐α protein levels (Figures 3R–U), and *IL‐1β*, *IL‐6*, *TNF‐α*, *MMP3* and *CXCL5* mRNA levels (Figure 3V–Z; Supporting Information Figure S5I–M) were found to be reduced in HFD‐fed *p16^−/−^* (or *ApoE^−/−^p16^−/−^*) mice and HFD+ABT263‐treated WT (or *ApoE^−/−^*) mice compared with WT (or *ApoE^−/−^*) mice fed a HFD. Taken together, our findings suggested that *p16* knockout and ABT263 treatment alleviated inflammaging in the lungs of mice fed a HFD.

### 3.4 Transcriptomics reveal promotion of the pro‐inflammatory response and metabolic reprogramming by p16 in the lungs of mice fed a HFD

**FIGURE 4:**
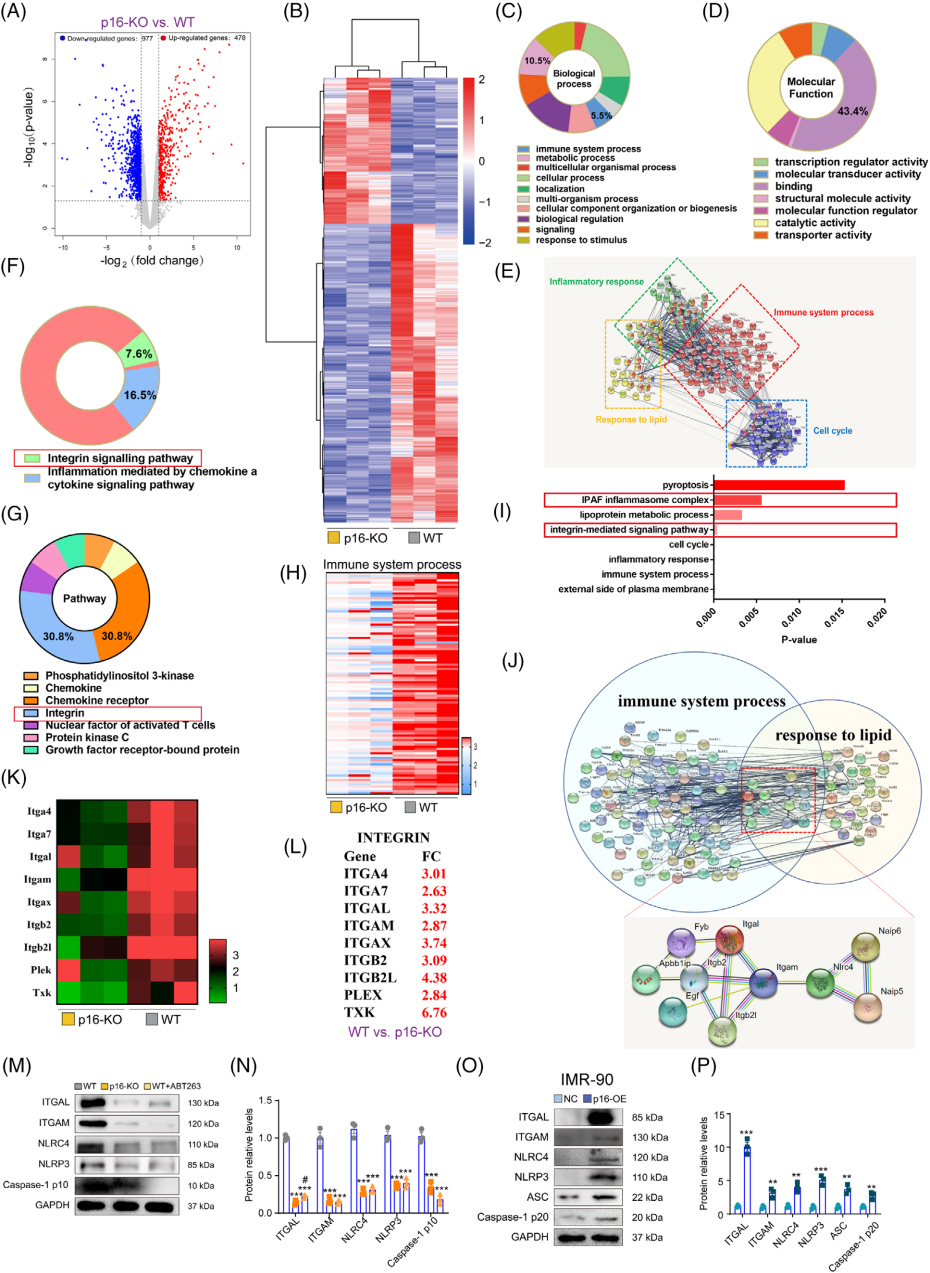
Transcriptomics reveal promotion of the pro‐inflammatory response and metabolic reprogramming by p16 in the lungs of HFD‐fed mice. (A) RNA‐seq analysis on HFD‐induced lungs (n = 3) identified 1455 statistically significant (*p* < .05) differentially expressed genes (DEGs) which are presented as a volcano plot: fold change from *p16^−/−^* (*p16‐*KO) mice versus WT mice. (B) Heatmap showing the DEGs identified by RNA‐seq analysis. (C) Functional profiling by the PANTHER Classification System was used to classify the DEGs according to ‘Biological Processes.’ (D) Functional profiling by the PANTHER Classification System was used to classify DEGs according to their ‘Molecular Function.’ (E) Detailed analysis of DEGs associated with the immune system and metabolic processes was carried out using the PANTHER Classification System. (F) Panther Classification System showing the Pathways of the immune system processes. (G) Heatmap showing the DEGs associated with the ‘Immune System.’ (H) Representative Protein‐protein interaction (PPI) network of the down‐regulated DEGs was constructed using STRING. Further functional analysis was performed with DAVID and significantly enriched categories are highlighted. (I) GO analysis showing up‐regulated DEGs between WT and *p16* ^−/−^ mice. (J) Genes related to ‘immune system processes’ and ‘response to lipid’ were merged in the PPI network using STRING. (K,L) Heatmap showing the mRNA expression levels of integrin pathway‐associated genes (*Itga4*, *Itga7*, *Itgal*, *Itgam*, *Itgax*, *Itgb2*, *Itgb2l*, *Plek* and *Txk*) in the lungs of 18‐month‐old HFD‐fed WT and *p16* ^−/−^ mice. Fold changes in the down‐regulated genes are shown in red. (M) Representative western blot showing ITGAL, ITGAM, NLRC4, NLRP3 and caspase‐1 p10 protein expression levels in the lung tissue of 18‐month‐old HFD‐fed WT, *p16* ^−/−^ and WT+ABT263. GAPDH was used as the loading control. (N) Protein bands were quantified by densitometric analysis and normalized to GAPDH levels. n = 3 biological replicates experiment. Values are presented as mean ± SEM, *p < .05, ***p < .001 compared with WT mice; ^#^ *p* < .05 compared with *p16*‐KO mice. Statistical analysis was performed using one‐way ANOVA. (O) IMR‐90 cells were transduced with *p16*‐overexpressing adenovirus, and treated with P&O to induce steatosis. Representative western blot showing ITGAL, ITGAM, NLRC4, NLRP3, ASC and caspase‐1 p10 protein expression levels. GAPDH was used as the loading control. (P) Protein bands were quantified by densitometric analysis and normalized to GAPDH levels. n = 3 biological replicates per experiment. Values are given as mean ± SEM. **p < .01, ***p < .001 compared with negative control (NC) group. Statistical analysis was performed with unpaired Student's *t*‐test.

To determine the role of p16 in HFD‐induced accumulation of senescent cells, RNA‐seq analysis was performed on lung tissue samples obtained from HFD‐fed 18‐month‐old WT and *p16* ^−/−^ mice to identify differentially expressed genes (DEGs) after *p16* knockout. RNA‐seq and bioinformatics analyses showed that 1455 genes were modified after p16 deletion (Figure 4A and Supporting Information Figure S6A–C). Compared with HFD‐fed WT mice, 478 of these genes were up‐regulated and 977 genes were down‐regulated in HFD‐fed *p16* ^−/−^ mice (Figure 4B). Analysis using the PANTHER classification system revealed that the down‐regulated DEGs were mainly associated with ‘immune‐inflammatory responses’ and ‘cell metabolism,’ suggesting that *p16* deletion may regulate these processes in the lung (Figure 4C). Molecular function analysis showed that these DEGs may be involved in intracellular transcriptional changes (Figure 4D). In addition, we analysed the protein interaction networks of these DEGs, and found that *p16* deletion affected ‘immune system processes,’ ‘inflammatory responses,’ ‘response to lipids,’ and the ‘cell cycle’ in pulmonary tissues of mice fed a HFD (Figure 4E). Analysis of the PANTHER gene list and gene ontology (GO) enrichment analyses indicated that after *p16* knockout, the integrin signalling pathway was significantly enriched in two key clusters that play important roles in inflammation and metabolism (lipogenesis) (Figure 4F and G). The genes identified by RNA‐seq analysis as being associated with immune system processes are shown in the Heatmap (Figure 4H). GO enrichment analysis revealed that the processes ‘integrin signalling’ and ‘inflammasome signalling’ were significantly enriched (Figure 4I). We then constructed a protein–protein interaction network map of immune‐inflammatory response and lipid metabolism‐related molecules, and found that integrin and inflammasomes were in the interaction areas for two key clusters (Figure 4J). Pokharel et al. previously demonstrated that 25‐hydroxycholesterol activates the integrin signalling pathway and exerts significant pro‐inflammatory effects.^30^ Thus, the activation of integrin in our HFD‐fed mice may be important during chronic inflammation. RNA‐seq analysis revealed downregulation of integrin pathway genes (*ITGA4*, *ITGA7*, *ITGAL*, *ITGAM*, *ITGAX*, *ITGB2*, *ITGB2L*, *PLEX* and *TXK*) and inflammasome pathway genes (*NLRC4*, *NAIP5* and *NAIP6*) after *p16* deletion (Figure 4K and L). These results suggested that *p16* knockout inhibited the interaction between the integrin and inflammasome signalling pathways. Thus, p16 may regulate the immune‐inflammatory processes and lipid metabolism in the lungs of mice fed a HFD.

Analysis of down‐regulated DEGs using the PANTHER classification system showed that *p16* knockout regulated immune‐inflammatory events and cell metabolism in the lungs of 18‐month‐old mice (Supporting Information Figure S6D and E). In addition, the expression of Toll‐like receptor (TLR) signalling pathways (*TLR7, TLR8*), the chemokine CCR family (*CCR3, CCR5, CCR7*) and *CXCL9*, which are closely related to SASP activation, was significantly reduced after *p16* knockout (Supporting Information Figure S6F). These results were consistent with our above‐described findings. As a key regulatory protein in cellular senescence, p16 mainly acts on CDK4/6 to induce cell cycle arrest. Our RNA‐seq data demonstrated that *p16* knockout affected the expression of cell‐cycle‐related genes (Supporting Information Figure S6G). This may explain the decrease of senescent cells in the lungs after *p16* knockout.

### 3.5 *P16* deletion or ABT263 treatment inhibits activation of the integrin‐inflammasome pathway and cellular glycolysis in lungs of mice fed a HFD

Next, we sought to determine whether *p16* deletion or ABT263 treatment could inhibit integrin‐inflammasome pathway in the lungs from aging HFD‐fed WT, *ApoE* ^−/−^, *p16^−/−^* and *ApoE^−/−^p16^−/−^* mice. Our immunohistochemical data indicated a significant reduction in cells positive for ITGAL, ITGAM, NLRP3, NLRC4, caspase‐1 and ASC in HFD‐fed *p16^−/−^* (or *ApoE^−/−^p16^−/−^*) mice and HFD+ABT263‐treated WT (or *ApoE^−/−^*) mice compared with WT (or *ApoE^−/−^*) mice fed a HFD (Supporting Information Figure S7A–P). Furthermore, a significant decrease in ITGAL, ITGAM, NLRC4 and caspase‐1 p10 protein levels, and *ITGAM*, *ITGAL*, *NLRC4, ITGB2*, *ITGB2L*, *TXK*, *NAIP5* and *NAIP6* mRNA levels also showed in *p16^−/−^* (or *ApoE^−/−^p16^−/−^*) mice fed a HFD, and HFD+ABT263‐treated WT (or *ApoE^−/−^*) mice compared with WT (or *ApoE^−/−^*) mice fed a HFD (Figure 4M and N, Supporting Information Figures S7Q and R, and S8).

We then asked whether p16 could up‐regulate the integrin‐inflammasome pathway during non‐inflammatory steatosis in vitro. Overexpression of p16 resulted in an increase in ITGAL, ITGAM, NRC4, NLRP3, ASC and caspase‐1 p20 protein levels in P&O‐induced IMR‐90 and in A549 cells compared with the negative control (NC), suggesting that p16 may activate the integrin‐inflammasome signalling pathway in P&O‐induced lung fibroblasts and alveolar epithelial cells (Figure 4O and P and Supporting Information Figure S7S and T).

**FIGURE 5:**
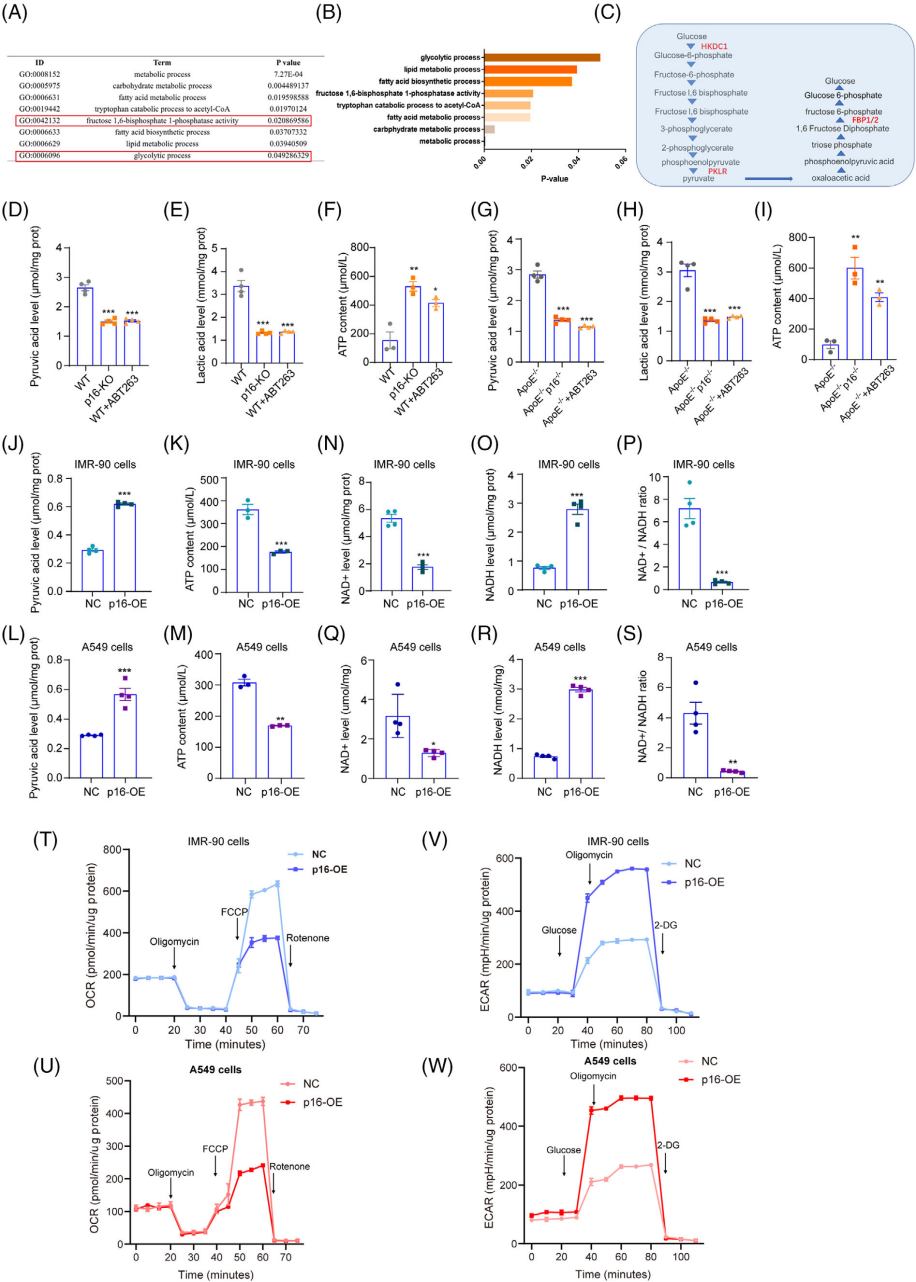
*P16* deletion decreases glycolysis in the lungs of HFD‐fed mice and in an in vitro model of non‐inflammatory steatosis in IMR‐90 and A549 cells. Functional analysis of the up‐regulated DEGs between WT and *p16^−−^* mice was performed using DAVID. Significantly enriched categories are highlighted. (B) Genes associated with glucose metabolism that were identified by RNA‐seq analysis. (C) Diagram showing the genes involved in the regulation of the glycolysis metabolic pathway. (D) Pyruvic acid levels, (E) lactic acid levels and (F) the ATP content in the lung tissue of 18‐month‐old WT, *p16* ^−/−^ and WT+ABT263 mice were assessed by colorimetry. Values are presented as mean ± SEM. *p < .05, **p < .01, ***p < .001 compared with WT mice. Statistical analysis was performed using one‐way ANOVA. (G) Pyruvic acid levels, (H) lactic acid levels and (I) the ATP content in the lung tissue of 18‐month‐old *ApoE^−−^*, *ApoE^−−^p16^−−^* and *ApoE^−−^* +ABT263 mice were detected by colorimetry. Values are given as mean ± SEM. **p < .01, ***p < .001 compared with *ApoE^−−^* mice. Statistical analysis was performed using one‐way ANOVA. IMR‐90 cells and A549 cells were transduced with *p16*‐overexpressing adenovirus, and treated with P&O to induce steatosis. (J–M) Pyruvic acid levels and ATP content were detected in these cells by colorimetry. (N–S) NAD^+^ levels, NADH levels and the NAD^+^/NADH ratio were measured. Values are presented as mean ± SEM. *p < .05, **p < .01, ***p < .001 compared with negative control (NC) group. Statistical analysis was performed using unpaired Student's *t*‐test. (T,U) The oxygen consumption rate (OCR) was measured in *p16*‐overexpressing IMR‐90 or A549 cells treated with P&O to induce steatosis. (V‐W) The extracellular acidification rate (ECAR) was measured in *p16*‐overexpressing IMR‐90 or A549 cells treated with P&O to induce steatosis.

Metabolic reprogramming in senescent cells is a critical event in inflammaging.^31^ Here, we found that the levels of enriched glycolytic‐related genes significantly reduced in HFD‐fed *p16^−/−^* mice compared with WT mice fed a HFD, suggesting that *p16* deletion reduced the glycolytic capacity of HFD cells (Figure 5A and B). In addition, hexokinase (HKDC), pyruvate kinase L/R (PKLR), and fructose‐1,6‐bisphosphatase (FBP) were identified as key metabolic enzymes that may affect cellular glycolysis in a p16‐mediated manner (Figure 5C). We also found that p16 knockout and ABT263 administration inhibited the expression of *HKDC1, DGKG, DGKB, FBP1, FBP2, PKLR* and *GCK* (Supporting Information Figure S9A–N).

Next, we examined whether *p16* deletion or ABT263 administration could inhibit metabolic reprogramming and reduce the glycolytic capacity in the lungs of aging HFD‐fed WT, *ApoE* ^−/−^, *p16^−/−^* and *ApoE^−/−^p16^−/−^* mice. We found a significant reduction in pyruvic acid and lactic acid levels together with a significant increase in ATP content in the lungs of HFD‐fed *p16^−/−^* (or *ApoE^−/−^p16^−/−^*) and HFD+ABT263‐treated WT (or *ApoE^−/−^*) mice compared with WT (or *ApoE^−/−^*) mice fed a HFD (Figure 5D–I). The effects of p16 on metabolic reprogramming and glycolytic capacity were also examined in vitro in p16‐overexpressing IMR‐90 and A549 cells treated with P&O to induce steatosis. A significant increase in pyruvic acid levels and reduction in ATP content was observed in P&O‐treated IMR‐90 and A549 cells overexpressing p16 compared with the NC, suggesting that p16 is involved in inhibiting metabolic reprogramming and reducing glycolytic capacity (Figure 5J–M). Enhanced glycolysis promotes activation of the inflammasome by increasing mitochondrial oxygen‐free radicals and disrupting the NAD^+^/NADH ratio. Previous studies have shown that treatment with 2‐deoxy‐D‐glucose, a glycolysis inhibitor, repressed activation of NLRP3 inflammasome.^32^, ^33^ Here, we found a significant reduction in NAD^+^ levels and the NAD^+^/NADH ratio, together with a significant increase in NADH levels in P&O‐treated IMR‐90 (Figures 5N–P) and A549 (Figures 5Q–S) cells overexpressing p16 compared with the NC. In vivo, we found that depletion of p16 caused an obvious increase in NAD^+^ levels and the NAD^+^/NADH ratio, while NADH levels decreased in the lungs of HFD‐fed *p16^−/−^* (or *ApoE^−/−^p16^−/−^*) and HFD+ABT263‐treated WT (or *ApoE^−/−^*) mice compared with WT (or *ApoE^−/−^*) mice fed a HFD (Supporting Information Figure S10).

Several studies have shown that lactic acid metabolism, induced by enhanced activity of pyruvate kinase, significantly promotes the production of IL‐1β and activation of NLRP3 inflammasomes.^33^ Since lactic acid is mainly produced by anaerobic metabolism in cells, we hypothesized that *p16* may affect the balance between anaerobic and aerobic metabolism. So, the oxygen consumption rate (OCR) and extracellular acidification rate (ECAR) in P&O‐treated IMR‐90 and A549 cells overexpressing *p16* were measured. We found that p16 overexpression promoted anaerobic respiration and glycolytic capacity, while inhibiting oxidative phosphorylation (OXPHOS), in IMR‐90 and A549 cells (Figure 5T–W).

Taken together, the above data suggest that p16 significantly inhibits OXPHOS, thereby promoting intracellular glycolysis in senescent cells.

### 3.6 HFD promotes activation of integrin‐inflammasome signalling pathway in physiologically aged mice

Next, we examined whether HFD promotes activation of integrin‐inflammasome signalling pathway in physiologically aged mice. We found a significant increase levels of SASP‐related genes (*CXCL5*, *IL‐6*, *MMP3*, *TNF‐α* and *IL‐1β*), aging‐related genes (*CDK1*, *CCNB2*, *CDKN1C* and *CCNB1*), and integrin‐inflammasome pathway genes (*ITGAM*, *ITGAL*, *NLRC4*, *ITGB2L*, *ITGB2*, *TXK*, *NAIP6* and *NAIP5*) in the pulmonary tissue of aged mice fed a HFD compared to aged mice fed a normal diet (Supporting Information Figure S11A–D). Similarly, ITGAL, ITGAM, NLRC4, NLRP3, ASC, caspase‐1, cleaved‐IL‐1β, IL‐6 and TNF‐α protein levels obviously increased in HFD‐fed mice compared with mice fed a normal diet (Supporting Information Figure S11E and F).

### 3.7 P16 interacts with SGK1 and inhibits K48‐polyubiquitin‐dependent degradation of SGK1 via the NEDD4L–UbcH5 complex

**FIGURE 6:**
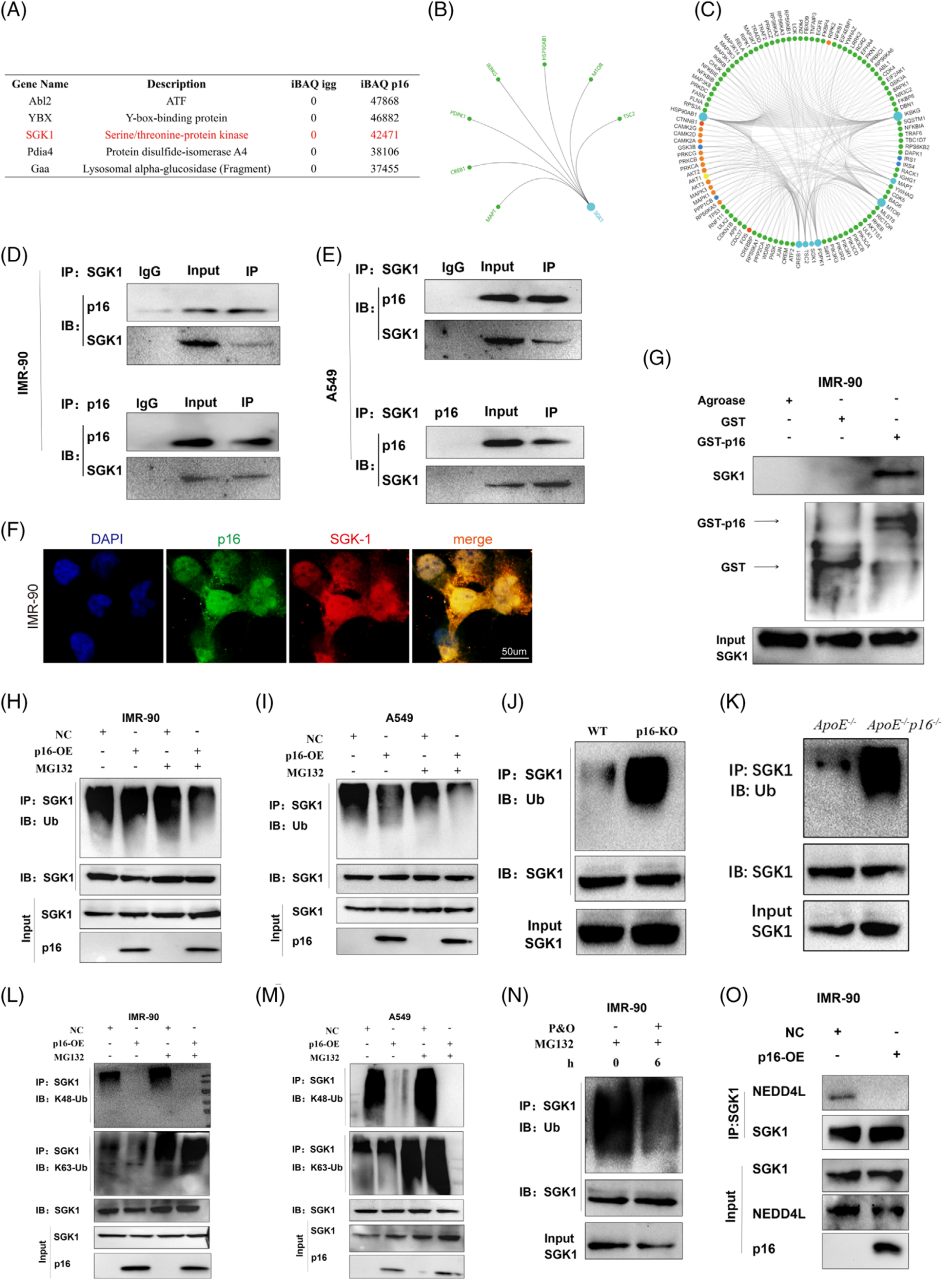
P16 interacts with SGK1 and inhibits K48‐polyubiquitin‐dependent degradation of SGK1 via the NEDD4L–UbcH5 complex. IMR‐90 cells were transduced with the Flag‐p16 overexpression adenovirus and treated with P&O for 24 h to induce steatosis. Co‐immunoprecipitation and mass spectrometry were used to identify proteins that interacted with p16. (A) Mass spectrometry analysis showing the proteins that interacted with p16 in mouse embryo fibroblasts after co‐immunoprecipitation. (B) GCBI software was used to identify proteins that interacted with SGK1. (C) GCBI software was used to identify potential downstream targets of SGK1. (D,E) IMR‐90 (D) and A549 (E) cells were transduced with Flag‐p16 overexpression adenovirus and treated with P&O for 24 h to induce steatosis. Co‐immunoprecipitation was performed using anti‐p16 or anti‐SGK1 antibodies to determine whether p16 and SGK1 interacted in vitro. (F) Representative immunofluorescence images showing the co‐localization of p16 and SGK1 in IMR‐90 cells. (G) The GST pull‐down assay was used to confirm the in vitro interaction between p16 and SGK1 in the IMR‐90 cell lysate. (H,I) An in vitro protein ubiquitination assay was performed to determine the ubiquitination levels of SGK1 in p16‐overexpressing, P&O‐treated IMR‐90 (H) and A549 (I) cells. (J,K) A protein ubiquitination assay was performed to determine the ubiquitination levels of SGK1 in the lung tissue lysates of 18‐month‐old, HFD‐fed WT (J), p16^−/−^ (J), ApoE^−/−^ (K) and ApoE^−/−^p16^−/−^ (K) mice. (L‐M) An in vitro ubiquitination assay was performed to determine the levels of K48‐ubquitination on SGK1 in p16‐overexpressing, P&O‐treated IMR‐90 (L) or A549 (M) cells. (N) in vitro ubiquitination assay in P&O‐treated IMR‐90 cells revealed that ubiquitination was induced in IMR‐90 cells for 6 h. (O) The co‐immunoprecipitation assay was used to determine the interaction levels between SGK1 and NEDD4L in control and p16‐overexpressing IMR‐90 cells.

**FIGURE 7:**
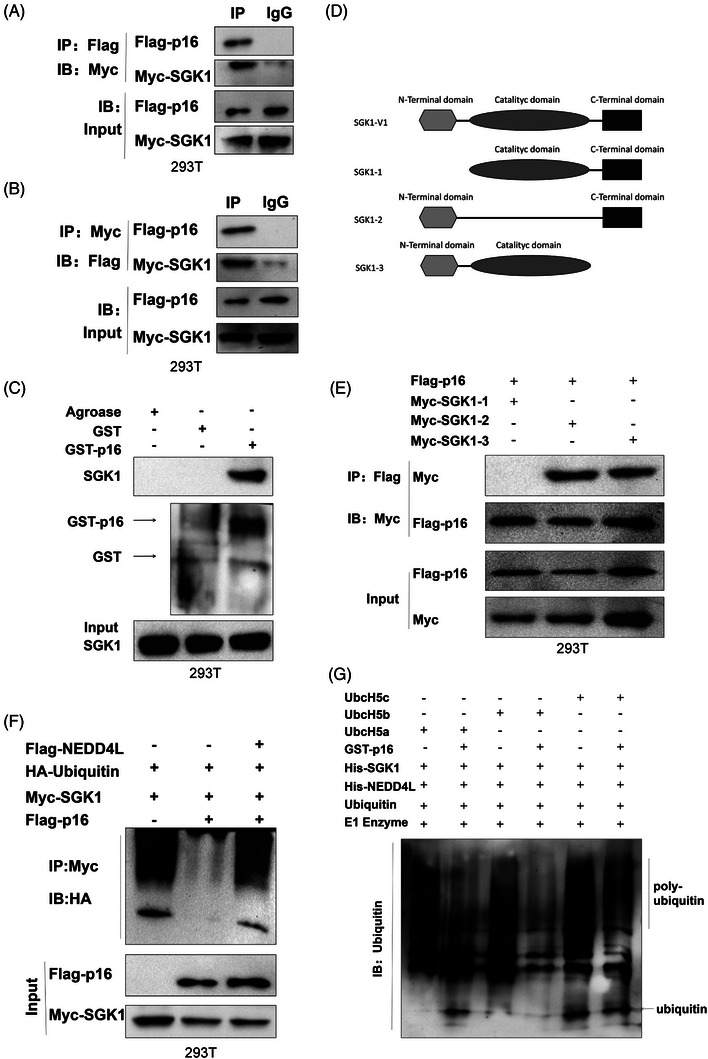
P16 interacts with the N‐terminal of SGK1 and inhibits ubiquitination of SGK1 mediated by the NEDD4L–UbcH5 complex. HEK293T cells were transfected with Flag‐*p16*, Myc‐*SGK1* and HA‐*Ubiquitin*, Flag‐NEDD4L and Myc‐WT‐*SGK1* or Myc‐mutated‐*SGK1* for 48 h. Cells were incubated with MG132 for 6 h. Cell lysates were collected and analysed. (A,B) Co‐immunoprecipitation was used to detect the interaction between SGK1 and p16. (C) GST pull‐down assay revealed an in vitro interaction between p16 and SGK1 in the cell lysate of 293T cells. (D) Diagram illustrating the structure of WT (SGK1‐V1) and three domain mutations (Myc‐SGK1‐1, Myc‐SGK1‐2 and Myc‐SGK1‐3) of SGK1. (E) Co‐immunoprecipitation was used to detect the interaction between the different domain mutated forms of SGK1 and p16. (F) A protein ubiquitination assay was used to examined the ubiquitination levels of SGK1 in NEDD4L‐overexpressing 293T cells. (G) in vitro ubiquitination assay was used to detect ubiquitination levels of SGK1.

Since p16 mainly exerts its regulatory role by interacting with other proteins via its INK domain, we performed co‐immunoprecipitation (co‐IP)/mass spectrometry analyses to identify the p16 interactome in mouse embryonic fibroblasts. We found that SGK1 interacted with p16 (Figure 6A). Bioinformatics analysis using the GCBI platform indicated that the proteins downstream of SGK1 (IKBKG, CREB1, PDPK1, MTOR and TSC2) regulated inflammation and metabolism (Figure 6B). The interaction network revealed associations between the cell cycle, inflammatory response, cell metabolism, proliferation and other signalling pathways (Figure 6C). Therefore, we hypothesized that p16 regulated HFD‐induced lung fibrosis via SGK1. Our co‐IP data revealed an interaction between p16 and SGK1 in IMR‐90 and A549 cells (Figure 6D and E) and 293T cells (Figure 7A and B). Immunofluorescence staining also revealed that p16 and SGK1 co‐localized in the cytoplasm of IMR‐90 (Figure 6F) and A549 (Supporting Information Figure S12A) cells. The results of our GST pull‐down assay with purified GST‐p16INK4a protein were consistent with the co‐IP and immunofluorescence findings (Figures 6, 7; Supporting Information Figure S12D). We then generated SGK1 protein constructs containing either the *N*‐terminal, kinase or C‐terminal domains to identify the specific domains through which p16 and SGK1 interacted. By transfecting HEK293T cells with plasmids containing mutations in the various SGK1 domains, we found that p16 bound specifically to the *N*‐terminal domain of the SGK1 molecule (Figure 7D and E). Previous studies have shown that the *N*‐terminal domain is closely associated with the ubiquitination and degradation of SGK1.^34^ Here, we found that overexpression of p16 resulted in increased expression of SGK1 in steatosis‐induced IMR‐90 cells (Supporting Information Figure S12B). We then examined whether p16 had a role in mediating the ubiquitination of SKG1 by treating IMR‐90 and A549 cells with cycloheximide and detecting the half‐life of SGK1 by western blot. We found that overexpression of p16 prolonged the half‐life of SGK1 (Supporting Information Figure S12E and F), suggesting that p16 may affect the ubiquitination and degradation of SGK1. Indeed, overexpression of p16 was found to reduce SGK1 ubiquitination levels in IMR‐90 and A549 cells (Figure 6H and I). In addition, we found that overexpression of p16 led to a reduction in K48‐linked poly‐ubiquitination of SGK1, but had no significant effect on K63‐linked poly‐ubiquitination levels (Figure 6L and M).

Our in vivo immunoprecipitation data revealed that SGK1 ubiquitination levels obviously increased in the lungs of HFD‐fed *p16^−−^* and *ApoE^−−^* mice than WT mice (Figure 6J and K). In addition, our co‐IP data showed that SGK1 ubiquitination levels were significantly reduced 6 h after the induction of steatosis in IMR‐90 and A549 cells (Figure 6N and Supporting Information Figure S12G). This may be due to high levels of p16 in steatosis‐induced cells (Supporting Information Figure S1D–F). Moreover, K48‐linked poly‐ubiquitination levels of SGK1 were significantly decreased in steatosis‐induced IMR‐90 and A549 cells (Supporting Information Figure S12J and K), while K63‐linked poly‐ubiquitination levels were not affected (Supporting Information Figure S12H–I).

The *N*‐terminal domain of SGK1 contains important nuclear localization sequences, and is recognized by the E3 ubiquitin ligase NEDD4L, which ubiquitinates and degrades SGK1.^34^ Therefore, we next asked whether p16 interfered with the binding of NEDD4L to SGK1. We found that p16 overexpression obviously reduced the interaction between SGK1 and NEDD4L in IMR‐90 and A549 cells (Figures 6O and Supporting Information Figure S12L). Furthermore, overexpression of NEDD4L significantly reduced p16‐mediated inhibition of SGK1 ubiquitination and degradation in HEK293T cells (Figure 7F). Together, our findings indicated that NEDD4L and p16 expression levels were negatively correlated in the regulation of SGK1 ubiquitination and degradation.

Previous studies have shown that NEDD4L recognizes and degrades downstream proteins predominantly through the HECT domain of the UbcH5 E2 ubiquitin ligase family of proteins.^35^ Thus, we next used purified NEDD4L, an SGK1 recombinant protein, and three E2 ubiquitin ligase proteins (UbcH5a, UbcH5b and UbcH5c) to assess the ubiquitination of SGK1 in vitro. We found that overexpression of p16 reduced NEDD4L‐mediated ubiquitination and degradation of SGK1. Similar results were obtained using the three E2 ubiquitin ligases UbcH5a, UbcH5b and UbcH5c (Figure 7G), indicating that p16‐mediated inhibition of SGK1 ubiquitination and degradation by NEDD4L was independent of a specific E2 ubiquitin ligase. Taken together, our findings demonstrated that p16 weakened the NEDD4L‐mediated K48‐polyubiquitin‐dependent degradation of SGK1, and that this process was upstream of the E2 ubiquitin ligase.

### 3.8 The SGK1 specific inhibitor EMD638683 or SGK1 siRNA ameliorates HFD induced‐pulmonary fibrosis

**FIGURE 8:**
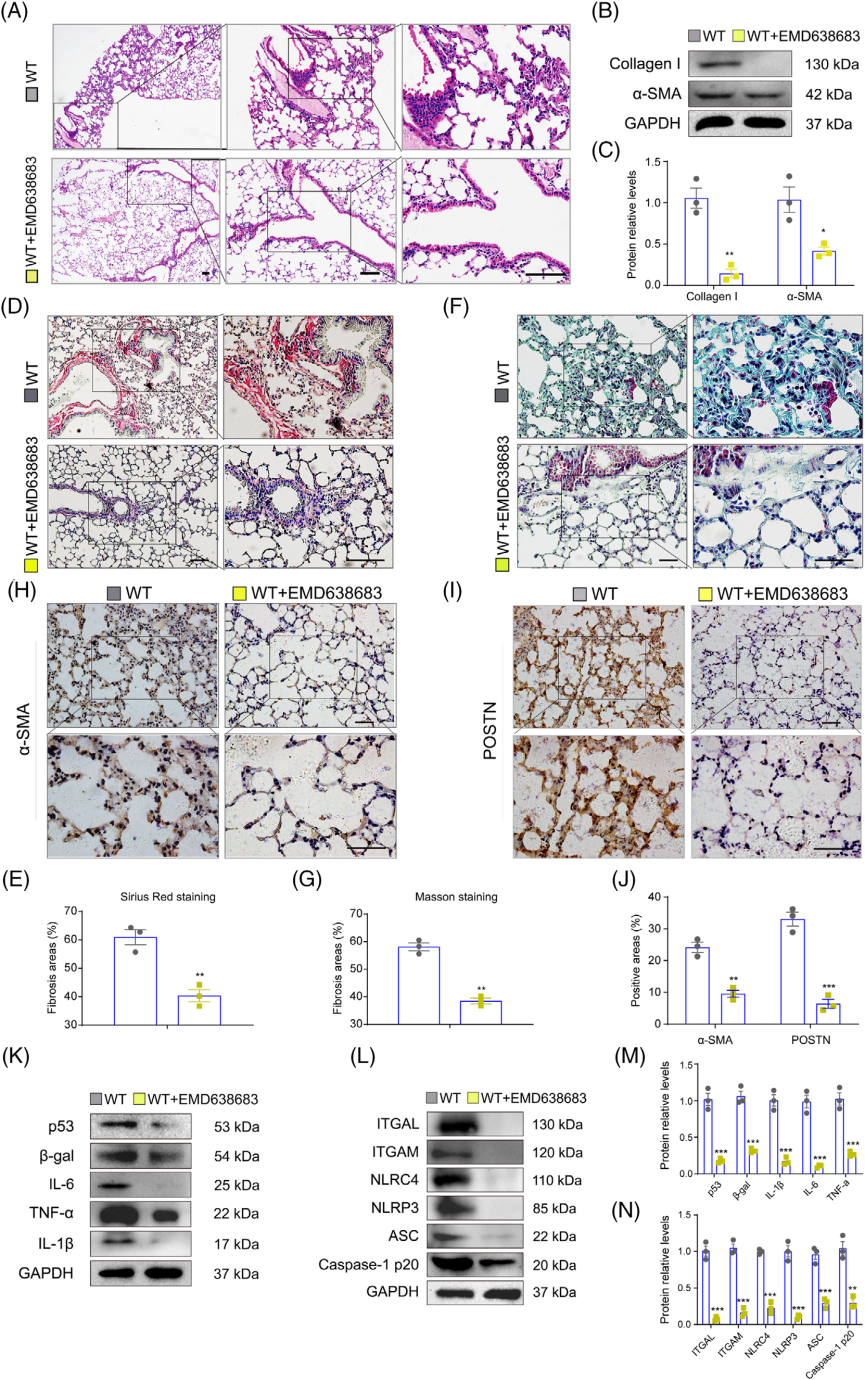
The SGK1 specific inhibitor EMD638683 ameliorates HFD‐induced pulmonary fibrosis. Twelve‐month‐old male WT mice were fed a HFD, and administered EMD638383 (i.p. daily, 20 mg per kg body weight) for 6 months. Lung tissues were collected for further analysis. (A) Representative H&E‐stained image showing cellular infiltration into the lung. (B) Representative western blot showing collagen 1 and α‐SMA protein expression levels in the lung tissue. GAPDH was used as the loading control. (C) Protein bands were quantified by densitometric analysis and normalized to GAPDH levels. n = 3 mice per group. Values are given as mean ± SEM, *p < .05, **p < .01 compared with WT mice. Statistical analysis was performed using unpaired Student's *t*‐test. (D,E) Representative images and statistical analysis of Sirius Red staining to assess collagen deposition in the lung tissue. (F,G) Representative images and statistical analysis of Masson's trichrome staining to evaluate collagen deposition in the lung tissue. (H‐J) Representative immunohistochemical images and statistical analysis of α‐SMA and POSTN in the lung tissue. (K‐L) Representative western blots showing p53, β‐gal, IL‐6, TNF‐α, IL‐1β, ITGAL, ITGAM, NLRC4, NLRP3, ASC and caspase‐1 p20 protein expression levels in the lung tissue. GAPDH was used as the loading control. (M,N) Protein bands were quantified by densitometric analysis and normalized to GAPDH levels in K and L, respectively. n = 3 biological replicates per experiment. Values are presented as mean ± SEM. *p < .05, **p < .01, ***p < .001 compared with WT mice. Statistical analysis was performed using unpaired Student's *t*‐test.

Since p16 exacerbated HFD‐induced pulmonary fibrosis via the regulation of SGK1, we next asked whether inhibition of SGK1 could rescue this process. Twelve‐month‐old mice were fed a HFD and administered EMD638683 (20 mg/kg) intragastrically for 6 months. H&E staining showed that EMD638683 significantly decreased exudation and inflammatory cell infiltration (Figure 8A), while Masson's trichrome and Sirius Red staining demonstrated that EMD638683 reduced pulmonary fibrosis and deposition of collagen fibres in the lung (Figure 8D and E). Immunohistochemical staining and immunoblotting revealed that EMD638683 inhibited collagen I and α‐SMA expression (Figure 8B,C,F and G), suggesting that EMD638683 alleviated HFD‐induced pulmonary fibrosis. We also found that EMD638683 reduced the levels of p53, β‐gal and p19 (Figure 8K–N and Supporting Information Figure S13A and B), as well as SASP factors including IL‐1β, IL‐6 and TNF‐α (Figure 7K and L and Supporting Information Figure S13C–H). Furthermore, ITGAL, ITGAM, NLRP3, NLRC4, ASC and caspase‐1 levels decreased by EMD638683 treatment (Figure 8M and N and Supporting Information Figure S13I–R), suggesting that EMD638683 can inhibit activation of the integrin‐inflammasome pathway.

Next, we sought to determine whether inhibition of SGK1 could inhibit p16‐mediated activation of the integrin‐inflammasome pathway during non‐inflammatory steatosis in vitro. EMD638683 was found to inhibit p16‐mediated activation of the integrin‐inflammasome pathway in IMR‐90 cells. Specifically, overexpression of p16 was shown to increase ASC, caspase‐1, cleaved‐IL‐1β, IL‐6, ITGAL, ITGAM, NLRC4 and NLRP3 protein expression levels in P&O‐treated IMR‐90 cells compared to the NC, while treatment with EMD638683 decreased the expression levels of these proteins (Supporting Information Figure S14).

Finally, the IMR‐90 and A549 cell lines were induced with *p16* overexpression adenovirus and transfected with small interfering RNA (si‐SGK1) targeting to SGK1 in vitro. After treating with P&O for 24 h, cell lysates were collected for further immunoblotting detection. The results found that after treating with si‐SGK1‐1 or si‐SGK1‐2, the protein levels of SGK1, NLRC4, NLRP3, ASC, caspase‐1 p10, IL‐1β, IL‐6 and TNF‐α decreased compared to *p16* overexpression group. We then detected protein levels of ITGAL and ITGAM and found that si‐SGK1 treatment inhibited levels of ITGAL and ITGAM. These results demonstrated that knockdown of *SGK1* in A549 and IMR‐90 cell lines could inhibit up‐regulation of integrin‐inflammasome signalling caused by *p16* overexpression (Supporting Information Figure S15).

## 4 DISCUSSION

Our study is the first to show that HFD‐induced accumulation of p16 leads to up‐regulation of SGK1 via inhibition of NEDD4L‐mediated K48‐linked poly‐ubiquitination and degradation of SGK1. P16‐mediated SGK1 accumulation promoted activation of the integrin‐inflammasome pathway and cellular glycolysis, increased secretion of SASP and aggravated accumulation of senescent cells and pulmonary fibrosis. Clearance of senescent cells following treatment with ABT263 or SGK1‐ specific inhibitor EMD638683 ameliorated HFD‐induced pulmonary fibrosis and reduced SASP. Our findings highlight the close relationship between HFD‐induced pulmonary fibrosis and senescent cells, and indicate that EMD638683 or ABT263 could be developed as potential drugs for the clinical treatment of pulmonary fibrosis in obese patients.

Obesity‐related respiratory diseases are closely associated with chronic pulmonary fibrosis.^15^ The obesity rate of IPF patients is twice as high as that of healthy people. Several lines of evidence have confirmed that hyperlipidaemia can lead to pulmonary interstitial fibrosis in IPF patients.^5^, ^36^ In addition, within 1 year, the mortality rate of obese IPF patients with a BMI of 30 kg/m^2^ was 1.71 times higher than that of non‐obese IPF patients.^37^ Furthermore, the low cardiopulmonary reserve capacity in obese people infected with COVID‐19 reportedly increases the severity of pulmonary manifestation.^38^ However, the detailed mechanisms regulating the relationship between obesity and lung fibrosis remain unclear. Previously, we demonstrated that aging‐related pulmonary fibrosis was correlated with the IL‐11 signalling pathway in prematurely aging mice.^23^ However, a major limitation of this previous study was that our findings were not based on physiologically aging mice. In contrast to our previous report, the current study did not show a critical role for IL‐11 in HFD‐induced pulmonary fibrosis, but instead identified a novel regulatory role for p16 in maintaining the stability of SGK1. Previous studies have reported that HFD‐induced pulmonary fibrosis was associated with high‐lipid‐induced chronic inflammation via neutrophil infiltration and overexpression of inflammatory factors including TNF‐α and IL‐1β.^13^, ^39^ Consistent with these findings, our study demonstrated significant changes in inflammaging in the HFD‐induced lung, with increased expression of classical SASP.

ABT263 has previously been shown to possess senolytic and anti‐fibrotic properties. Treatment with ABT263 can induce apoptosis in aging pulmonary myofibroblasts and type II alveolar epithelial cells by inhibiting Bcl‐2/xl, as well as reverse changes in mouse pulmonary fibrosis caused by ionizing radiation.^40^ Moreover, ABT‐263 can ameliorate cardiac fibrosis,^41^ renal fibrosis,^42^ liver fibrosis^43^ and skin fibrosis^44^ through clearing senescent cells, reducing SASP and downregulating TGF‐β signalling. Here, we found that ABT263 ameliorated HFD‐induced pulmonary fibrosis and reduced SASP.

Elimination of senescent cells by *p16* deletion or ABT263 treatment resulted in inhibition of inflammaging and reduced SASP. Interestingly, our RNA‐seq data revealed that HFD‐induced accumulation of senescent cells and SASP were mainly correlated with activation of the integrin‐NLRC pathway. Previous studies in intestinal epithelial cells demonstrated a critical role for integrins in activating the downstream NLRP3 signalling pathway after *Yersinia* infection.^45^ Here, we showed that p16 promoted activation of NLRC4 and NLRP3 and their downstream targets, ASC and caspase‐1 via the integrin signalling pathway. This finding was different from that observed in prematurely aging mice. Previously, free fatty acids have been found to increase ATF‐4 and CHOP expression levels and pressure in the endoplasmic reticulum (ER).^13^ ER stress leads to cell senescence and activation of the inflammasome.^46^ ER stress could activate transcription factor 4/p16 signalling in senescent renal tubular epithelial cells.^47^ Thus, we hypothesized that p16 may affect activation of the integrin‐inflammasome pathway via ER stress in the HFD‐induced lung.

Metabolic reprogramming in senescent cells is another recently discovered process that affects inflammaging.^26^ Several studies have reported the critical role of glycolysis in IPF‐associated fibrosis.^27^, ^28^ Consistent with our findings, the accumulation of senescent cells has previously been associated with decreased NAD^+^ levels in tissues.^48^ Here, we found that HFD caused a reduction in NAD^+^ levels and increase in NADH^+^ levels, while *P16* knockout or ABT263 treatment reversed this phenotype. Excessive cellular glycolysis and impaired OXPHOS may have critical roles in inflammaging. We found that *p16* overexpression significantly induced a higher capacity of cellular glycolysis and inhibited OXPHOS in mouse lung tissue and cells. Previous studies have shown that an imbalance between OXPHOS and glycolysis in microglia aggravated LPS‐induced neuroinflammation.^49^ Furthermore, glycolysis has been shown to promote NLRP3 inflammasome activation through pyruvate kinase M2 (PKM2).^50^ Consistent with previous reports, in the current study p16 was found to promote an imbalance between OXPHOS and glycolysis in senescent fibroblasts and epithelial cells via SGK1. Furthermore, p16 up‐regulated expression of PKLR to affect cellular glycolysis. Therefore, we speculated that in senescent cells, p16 might also regulate activation of the NLRP3 inflammasome via metabolic remodelling. However, our RNA‐seq and protein‐protein interaction analyses indicated that the integrin‐inflammasome pathway also participated in metabolic reprogramming of senescent cells. Indeed, we found that the inflammasome and metabolic reprogramming had more complex inter‐modulatory networks in senescent cells. In addition, recent studies have demonstrated that NAD^+^ supplementation ameliorated inflammaging‐related diseases.^51^ However, further studies are required to determine whether NAD^+^ supplementation could rescue HFD‐induced lung fibrosis. In summary, our findings revealed critical roles and mechanisms of p16 in mediating metabolic remodelling in senescent cells.

SGK1, a member of the protein kinase subfamily, is a serine/threonine kinase with high homology to the second messenger. Previous study shows that SGK1 overexpression is associated with pulmonary fibrosis,^52^ cardiac fibrosis^53^ and renal fibrosis.^54^ SGK1 up‐regulated by TGF‐β1 in human lung fibroblasts could induce organic fibrosis, and further aggravated NF‐κB‐mediated pro‐inflammation via phosphorylating IκB.^55^ In addition, SGK1 could activate NLRP3‐inflammasome, and CGAS‐STING‐mediated pro‐inflammatory pathways in glia, and SGK1 inhibition rescues pro‐inflammatory effects.^56^ Furthermore, SGK1 plays a critical role in the pro‐glycolysis via regulating glucose uptake receptor‐GLUT1.^57^ Mechanically, SGK1 could up‐regulate expression of GLUT1 and increase the availability of glucose for glycolysis. SGK1 could also enhance Na^+^/H^+^ ions exchanger which generates an alkaline cytosolic pH, a pre‐requisite for an increase of glycolytic flux.^58^ Interestingly, this study revealed the critical role of SGK1 in p16‐ positive senescent cells, thus, exacerbating HFD‐induced pulmonary fibrosis. We also reported the role of p16 in inhibiting the NEDD4L ubiquitination complex and subsequent degradation of SGK1. SGK1 accumulation caused by p16 over‐expression activated integrin‐inflammasome and glycolysis pathways. Our study further enriched the role of SGK1 in fibrosis, and solidly supported the potential therapeutic value of SGK1 inhibition in pulmonary fibrosis.

Our study is the first to report interactions between SGK1 and p16, as well as a role for p16 in mediating SGK1 ubiquitination and degradation in senescent cells. We found that p16 interacted with *N*‐terminal domain of SGK1 and inhibited ubiquitination of SGK1. There are six ubiquitination sites (K4, K9, K29, K41, K50 and K59) in *N*‐terminal domain of SGK1. Thus, we speculated that p16 may affect the ubiquitination of these lysine sites at the *N*‐terminal domain of SGK1. Further studies are required to determine if this is the case.

Previously,^6^ bleomycin was shown to induce high p16 protein expression levels in fibroblasts and epithelial cells, while having no effect in endothelial cells. Thus, cell senescence in the mouse bleomycin‐induced IPF model occurs mainly in fibroblasts and epithelial cells. ABT263 was used to treat bleomycin‐induced IPF and cleared aging fibroblasts in the lung. However, it is unclear whether aging epithelial cells were also cleared.^6^ Significant accumulation of p16‐positive senescent cells has been observed in the lungs of IPF patients, with p16‐positive senescent cells widespread in lung fibroblasts and epithelial cells.^6^ Our results suggested that HFD could promote cell senescence in lung fibroblasts and alveolar epithelial cells. However, the impact on endothelial cells has not been evaluated in this study, and is a limitation of this article.

Although a key role for p53 has previously been reported in AT2 cell senescence in IPF, this single‐cell sequencing study did not show the key role of p16 in IPF‐mediated pulmonary fibrosis.^59^ Thus, it is possible that p53‐mediated cell senescence signalling plays an important role in HFD‐induced lung fibrosis. Moreover, our results showed that p53 and p21 levels were obviously increased in lungs of mice fed a HFD, suggesting that HFD‐induced accumulation of aging cells and pro‐fibrosis might be mediated by multiple senescence signalling pathways. At present, we cannot distinguish between the distinct roles of the different cell types (fibroblasts, type I and II alveolar epithelial cells) and their cross‐talk in this pathological process. Thus, in future studies, we will use single‐cell sequencing technology to address this issue. In addition, the construction of fibroblast or epithelial cell‐specific knockout mice would be beneficial in future studies.

Although we have observed some anti‐fibrotic effects in the mouse model through the specific small molecule EMD638683, p16‐mediated pulmonary fibrosis is complex and has multiple downstream signals. Previously, we described a key role for p16 in up‐regulating the TGF‐β1/IL‐11/MEK/ERK pathway in SAPF.^23^ Therefore, we believe that it may be more effective to design treatment interventions targeting p16 rather than its downstream molecules. With the gradual increase and wide application of mRNA drugs,^60^ the development of small nucleic acid drugs targeting p16 may have more advantages and expectations in clinical therapy in the future.

## 5 CONCLUSIONS

Our findings show that HFD induced accumulation of p16, which interacted with *N*‐terminal domain of SGK1, thereby interfering with the interaction between NEDD4L and SGK1, and subsequently inhibiting the NEDD4L–Ubch5‐mediated K48‐polyubiquitin‐dependent degradation of SGK1. SGK1 activated the integrin‐inflammasome pathway, while cellular glycolysis induced SASP and aggravated the accumulation of senescent cells and pulmonary fibrosis. ABT263 or EMD638683 may ameliorate these processes and could, therefore, act as potential drugs to treat pulmonary fibrosis and improve respiratory dysfunction in obese patients.

## CONFLICT OF INTEREST STATEMENT

The authors declare that they have no conflict of interest.

## Supporting information

Supporting Information

## Data Availability

The datasets presented in this study can be found in online repositories. The names of the repository/repositories and accession number(s) can be found below: http://www.ncbi.nlm.nih.gov/bioproject/SUB11359783/overview. The BioProject ID is PRJNA830843.
