# Correction to “Inflammasome activation and metabolic remodeling in p16‐positive aging cells aggravates high‐fat diet‐induced lung fibrosis by inhibiting NEDD4L‐mediated K48‐polyubiquitin‐dependent degradation of SGK1”

**DOI:** 10.1002/ctm2.70379

**Published:** 2025-07-24

Gu X, Meng H, Peng C, et al. Inflammasome activation and metabolic remodelling in p16‐positive aging cells aggravates high‐fat diet‐induced lung fibrosis by inhibiting NEDD4L‐mediated K48‐polyubiquitin‐dependent degradation of SGK1. *Clin Transl Med*. 2023;13(6):1308.

In this article, the authors have just realized the wrong usages of immunoblotting bands for β‐gal and p16 presented in Figure S1D and immunoblotting band for K48‐polyubiquitin (Ub) presented in Figure S12K. The corrected Figures S1D and S12K are as follows. The quantitative analysis in Figure S1F has been conducted using this accurate immunoblotting result of Figures S1D.

The authors sincerely apologize for these errors. This correction does not affect the study's conclusions.

## Supporting information



Supporting infomation

Supporting infomation

