# Supplementary material for: Inflammasome activation and metabolic remodelling in p16‐positive aging cells aggravates high‐fat diet‐induced lung fibrosis by inhibiting NEDD4L‐mediated K48‐polyubiquitin‐dependent degradation of SGK1
Source: Clin Transl Med. 2023 Jun 21;13(6):e1308. doi: 10.1002/ctm2.1308 (PMC10285269; doi:10.1002/ctm2.1308)
Supplement: Supplementary file 1 — Supporting Information [file CTM2-13-e1308-s001.docx]

**Supplemental data and materials**

**Complete Materials and Methods**

**Animals and Treatment**

To construct *p16* knockout mice, we deleted Exon 1α of the *p16* gene in mice. Male and female *p16* heterozygote (*p16^+/-^*) mice, that background was FVB N2, were crossed to produce *p16* knockout (*p16*KO, *p16^-/-^*) mice and WT littermates as described previously^1, 2^. Briefly, a neomycin-resistance cassette flanked by two loxP sites was cloned into an EcoRI ± Not I fragment at the XhoI and XmnI sites in exon 1α. These sites, which flanked the translational start site of *p16*, were destroyed in the targeted allele^3^. Western blot analysis revealed that although p16 protein was not detected in serially passaged MEFs (obtained from *p16^-/-^* mice), p19^ARF^ (in mouse) (homologous gene to human p14^ARF^) was detected^3^. *ApoE* knockout (*ApoE^-/-^*) mice were got from Nanjing University and mated with *p16^-/-^* mice, which were then mated to produce *ApoE^-/-^p16^-/-^* mice. Male *ApoE^-/-^p16^-/-^* mice were maintained on high-fat diet (HFD) until they reached 12 months of age, when they used in subsequent experiments. *ApoE^-/-^p16^-/-^* mice deficient in *ApoE* and *p16* were genotyped separately. All mice were bred in SPF animal conditions and housed in mouse rooms maintained at 23^o^C. In this experiment, we complied “Guide for the Care and Use of Laboratory Animals”. Our experiment was approved by Nanjing Animal Experimental Ethics Committee (Permit Number: IACUC-1809033).

Senescent cells were eliminated in *vivo* by treating mice with Navitoclax (ABT263), which was purchased from Selleck (Catalog Number: S1001; Selleck, Houston, TX, USA)^2, 4^. Briefly, mice were administered ABT263 intragastrically (i.g.) in per cycle lasting 7 days at a dose of 50mg/kg/d and lasted for 6 months. After one cycle ended, the mice got 14 days interval. SGK1 was inhibited *in vivo* by administering EMD638383 (HY-15193; MedChemExpress, Monmouth Junction, NJ, USA) i.p at dose of 20 mg/kg/d^5^.

**Cell culture and *in vitro* treatment**

IMR-90 and A549 cell lines were obtained from Zhong Qiao Biotechnology Co., Ltd and cultured in DMEM (Gibco, Waltham, MA, USA), contained fetal bovine serum (Gibco) at concentration of 10%.

To induce steatosis, cells were incubated with 1 mmol/L sodium palmitate and sodium oleate for 24h. SGK1 was inhibited *in vitro* by treating cells EMD638683 (10 μM) for 24h^6^.

The half-life of SGK1 was determined by treating cells with cycloheximide (HY-12320, MedChemExpress; 100 μg/mL)^7^ for different lengths of time. Cell lysates were collected and SGK1 levels were analyzed by immunoblotting.

**Plasmid construction and cell transfection**

This study utilized various plasmids from Shanghai Quanyang Co., Ltd. (Shanghai, China), including human Flag-p16, human Myc-SGK1, human Flag-NEDD4L, ubiquitin (HA-Tag), K48-ubiquitin (HA-Tag), and K63-ubiquitin (HA-Tag) plasmids. In order to further understand the structure and function of the SGK1 protein, researchers utilized information from the UniProt database (<https://www.uniprot.org/>) to reveal the composition of the SGK1 protein, which includes the N-terminal domain (amino acids 1 to 60), the kinase domain (amino acids 98 to 355), and the C-terminal domain (amino acids 356 to 431). Based on the structure of the human SGK1 protein, researchers commissioned Quanyang Company to construct three domain-mutated plasmids with Myc tags. Subsequently, researchers transfected these plasmids into HEK293T cell lines via Lipofectamine 3000 reagent (Invitrogen) following instructions. Further investigation of interactions and functions of the SGK1 protein was conducted through immunoblotting and co-immunoprecipitation (co-IP) experiments using the lysates of HEK293T cells.

**Lentiviral transduction**

Lentiviral particles overexpressing p16 and its Negative Control (NC) overexpression lentivirus were obtained from Hanbio Co., Ltd. (Shanghai, China) to investigate role of p16 in cellular processes. IMR-90 and A549 cells were transduced with the lentiviral particles at MOI of 1:100. Transduction was carried out for a duration of 24 hours to allow efficient delivery of the lentiviral particles into the target cells.

**HFD mouse model**

Twelve-month-old male mice with genotypes *ApoE^-/-^*, *p16^-/-^*, *ApoE^-/-^p16^-/-^*, and WT (wild-type) were selected as experimental subjects in this study. These mice were subjected to a HFD consisting of 60% fat content, provided by SYSE Bio-Tec (Changzhou, China), for a period of 6 months. The composition of the HFD and normal diet (ND) used for comparison is presented in **Table S1**. The mice were weighed on a weekly basis to monitor changes in body weight throughout the feeding period. Following the 6-month dietary intervention, mice were sacrificed, and collected lung tissue for subsequent analysis.

**Western blots analysis**

To extract total protein from lung tissues, RIPA buffer solution supplemented with proteolytic enzyme inhibitors was used in this study. The concentration of total protein was measured via BCA assay (Thermo Fisher Scientific). Tissue was fully homogenized at a frequency of 65 Hz and subsequently cooled for 15 minutes in a centrifuge with a capacity of 12,000 cubic grams. Then loading buffer was added and heated to 95-100 degrees Celsius for 5 minutes. The protein samples were collected for subsequent SDS-PAGE analysis and transferred onto PVDF membranes. The membranes were blocked in 5% nonfat dry milk powder for 1 hour. Primary antibodies were used at 4℃overnight. All antibodies in this research are listed in **Table S2**. Following three washes with water for 5 minutes each and treated with HRP-conjugated secondary antibodies (diluted at 1:3,000) at 25℃ for 1 hour. Membranes were visualized via ECL detection system and Bio-Rad imaging system (Bio-Rad Laboratory), and the obtained images were quantified using ImageJ software.

**Histological staining**

Paraffin-embedded tissue sections, 4 microns thick, were utilized in this study for the assessment of cellular infiltration using Harris modified hematoxylin staining (Harris Review hematoxylin, China, China, Sorabio). Collagen deposition in the lung tissue was evaluated through the application of the Marson trichrome method (Massonian Tridge) (Sigma, St. Louis, Missouri, USA) and the Sirius Red method (Solarbio). The area of trichromatic positive staining in the captured images was quantified using Fiji, an image processing software based on ImageJ. To detect senescence-associated (SA)-β-galactosidase activity. As previous described, cryosections of pulmonary tissue were stained with SA-β-galactosidase staining kit (Beyotime) following instructions. **In the representative graphs, the percentage of SA-β-galactosidase positive cells relative to total number of nuclei stained with nuclear fast red was quantified.**

Sections were subjected to immunohistochemical staining using monoclonal antibodies against α-smooth muscle actin, collagen I, or POSTN to assess the expression levels of profibrotic proteins. The procedure involved deparaffinization and rehydration of the sections in xylene and ethanol solutions, respectively. Antigen retrieval was performed by boiling the samples in PBS (0.01 mM, pH 7.4) for 15 minutes. Subsequently, endogenous peroxidases were deactivated by incubating the samples in 6% H_2_O_2_ for 20 minutes and blocked with 10% goat serum for 1 hour. Primary antibodies were then applied and incubated overnight at 4℃. Then washing in PBS solution, secondary antibodies were used to incubation for 1 hour at room temperature. Following another round of washing, the samples were incubated with Vectastain Elite ABC-HRP according to instructions. Staining was visualized by incubating the sections with the DAB chromogen, as previously described. The positive-stained areas were quantified using ImageJ software. Approximately 3-5 images were randomly captured at ×40 magnification, and **percentage of positive areas relative to total tissue areas was calculated as "XXX positive areas (%)". Furthermore, the percentage of positive cells relative to the total number of nuclei stained with hematoxylin was calculated to demonstrate "XXX positive cells (%)" in the representative graphs.** Details of antibodies used for immunohistochemical staining are provided in **Table S3**.

**CellEvent™ Senescence Green Flow Cytometry**

Primary fibroblasts or epithelial cells were isolated and cultured from 18-month-old mice that were either on ND or HFD, following the previously described protocol. Cells (4−5 × 10^5^) were washed with PBS and fixed at room temperature (RT) using 4% paraformaldehyde. Subsequently, CellEvent™ Senescence Green Probe (dilution 1:1,000) in CellEvent™ Senescence Buffer were applied and incubated for 90 minutes at 37ºC (#C10840; Invitrogen, Carlsbad, CA, USA). After washing with 1% bovine serum albumin (Sigma-Aldrich) and PBS, then subjected to flow cytometry analysis using a 488-nm laser in a FACScalibur flow cytometer (Becton Dickinson, Heidelberg, Germany). The acquired data were then analyzed to evaluate the senescence-associated fluorescence signals.

**Immunofluorescence staining**

Lung tissue samples were dehydrated using a gradient sucrose solution series of 20%, 30%, and 40%. Subsequently, samples were embedded in cutting temperature compound (#4583, Sakura Finetek USA Inc., CA, USA) and cryosectioned into 10-μm thick serial. Sections were blocked with 5% BSA for 1 hour. Primary antibodies were then applied and incubated at 4℃. Then sections were treated with Alexa Fluor Plus 488-conjugated anti-rabbit secondary antibodies and 594-conjugated anti-goat secondary antibodies (Jackson Laboratory) for 1 hour. Following three additional washing, sections were treated with 4',6-diamidino-2-phenylindole (DAPI, Sigma-Aldrich). Visualization of sections was performed using fluorescence microscope (Zeiss). **The percentage of positive cells relative to total number of DAPI-positive nuclei was calculated to quantify the "XXX positive cells (%)" in the representative field.** Details of antibodies for immunofluorescence staining can be found in **Table S4**.

**Quantitative real-time polymerase chain reaction (RT-qPCR)**

Total RNA was extracted from lung tissue via the TRIzol (ThermoFisher Scientific), following the previously protocol^8^. Reverse transcription was carried out according to the production manual using the first-generation gene synthesis kit of HiScrip III (Vazyme, China, R312-01, RiScrip, China). Real-time quantitative PCR (RT-qPCR) uses the FastStart ™SYBR green Mix suite (Sigma-Aldridge-Inc.) on Application Biological System 7300 (Foster, California, USA), as described by the manufacturer, on Application Biology System 7300 (Application Biology System 7300) Real-time PCR (RT-qPCR). GAPDH were used as normalized gene. The primer sequences used for the RT-qPCR reactions can be found in **Table S5**.

***In vitro* siRNA transfection**

**According to above process, siRNA (small interference RNA) for specifically inhibiting expression of SGK1 is prepared. The 60-70% of IMR-90 and A549 were cultured on a 6-well plate, and 50 nM (NC) or SGK1 siRNA was transfected into the group using Lipofectamine 3000 (Invitrogen) according to manufacturer's instructions. After 48 hours, the cell lysates for further determination were collected. The sequences of siRNAs targeting human SGK1 can be found in Table S6, and the siRNA sequences were reported previously**^10^.

**Co-immunoprecipitation (Co-IP) assay**

Co-immunoprecipitation (Co-IP) was performed using Pierce Classic Magnetic IP/Co-IP Kit (Pierce Biotechnology, USA) according to instructions. Briefly, HEK293T cells were transfected with plasmids containing specific SGK1 domain mutations, including the N-terminal domain (1 to 60 amino acids), Myc-SGK1-2 (kinase domain, 98 to 355 amino acids), and Myc-SGK1-3 (C-terminal domain, 356 to 431 amino acids). Cell lysates from IMR-90, A549, or HEK293T cells were incubated overnight at 4℃ with either the IgG control or specific precipitation antibodies, including p16 (Abcam, Freemont, CA, USA), SGK1 (Cell Signaling Technology, USA), Myc-Tag (CST), or DYKDDDDK Tag (CST). Co-IP or total cytoplasmic lysates were analyzed by western blotting. To minimize contamination from light and heavy antibody chains, Clean-Blot IP (Thermo Fisher Scientific) and antibodies derived from different animal species were utilized. The antibodies used in experiment are provided in **Table S2**.

**Co-IP and LC-MS/MS analysis**

As described previously^11^, IMR-90 cells were transduced with Flag-p16 or Flag-vector overexpression adenoviruses. To induce steatosis, cells were treated with P&O. Subsequently, samples were collected, lysed, and stored at -80 ℃. Protein samples were incubated with protein A/G magnetic beads (Thermo Fisher Scientific) and anti-Flag antibody (Abcam). After five washes of the magnetic beads with appropriate buffers for 5 minutes each, the supernatant was removed. For protein denaturation, 1× SDS loading buffer was added to the magnetic beads. According to previously described^12^, IP samples were analyzed by LC-MS/MS. Within 70 minutes of 5-45% (v/v) acetonitrile-0.1% (v/v) and formic acid solution (v/v), MS scan range of m/z 450-1500. MS/MS spectra were analyzed using MASCOT server searching the Arabidopsis Information Resource (TAIR8) database.

**GST pull-down assay**

GST-p16 plasmid was transfected into Escherichia coli (E. coli), and fusion protein was prepared according to the previously described protocol^13^. After that, fusion protein (GST) of 100 ug GST and GST-p16 was pre-equalized on 50 ug (USA) agarose beads (Millipore). Incubate the beads at 4℃ for 60 minutes, then rotate gently. The solutes of IMR-90 and A549 were then added to GST-p16 and GST. The resulting complex solution rotates slowly at 4℃ for overnight. The sample was then eluted (glutathione PBS of pH8.0,10 mM) for further immunoblotting analysis.

***In vivo* and *in vitro* ubiquitination assay**

A549 and IMR-90 cell lysates were incubated with MG132 (20 μM) in complete medium for 6 hours to inhibit proteasomal degradation. Co-IP experiments were performed to assess levels of ubiquitination, K48-linked ubiquitination, and K63-linked ubiquitination of SGK1. Furthermore, the ubiquitination levels of SGK1 were examined in lung tissue samples obtained from 18-month-old wild-type (WT), *p16^-/-^*, *ApoE^-/-^*, and *ApoE^-/-^p16^-/-^* mice. The lung tissue was fragmented into small pieces and homogenized using a Dounce homogenizer. The homogenized samples were centrifugated at 13,000 × g for 15 minutes in a pre-cooled centrifuge, and the supernatants were used to immunoprecipitation assays for analysis.

For the *in vitro* ubiquitination assay^14^, recombinant human p16 GST protein was obtained from Proteintech (Ag29567, city, China), recombinant human SGK1 His protein was obtained from Atagenix Laboratories (ATAP00261, city, China), recombinant human NEDD4L GST protein was obtained from Novus Biologicals (H00023327, USA), and the E2-Select Ubiquitin Conjugation Kit was obtained from Bio-Techne (K-982, city, China). Samples were incubated at 37°C for 6 hours in buffer complex containing 10× reaction buffer, 10× E1 enzyme, 5× ubiquitin, 10× Mg2^+^-ATP, 20× E2 enzyme, NEDD4L, SGK1, GST/GST-p16, and H_2_O to make a total volume of 20 μL. The final products were analysis by SDS-PAGE, and levels of ubiquitination were assessed by immunoblotting using ubiquitin antibodies.

**RNA-seq and bio-informatic analyses**

For the RNA-seq analysis, RNA was isolated from the lung tissue of 18-month-old HFD-fed p16^-/-^ knockout and WT male mice using a previously described method (Reference 15). The reaction buffer was synthesized by the first section of NEBNext, and the extracted RNA sample was dissolved at elevated temperature. A DNA sequence was amplified by M-MULV reverse transcriptase and random hexamer primers. Subsequently, the second strand dsDNA was generated and the fragmented residues were turned into a blunt end by exonuclease or polymerase hydrolysis. The obtained dsDNA was alkalized at the 3' end of the gland and then reacted with NEBNext to form a hairpin-like structure. Following purification with the AMPure XP system, DNA fragments ranging from 150 to 200 bp in length were obtained. The DNA fragments were subjected to sequencing using the HiSeq 2500 sequence. DEGs were identified based on fold expression changes calculated. The sequencing quality of the trimmed sample data was assessed in FastQC. The DEGs were normalized using the transfer matrix method (TMM). DEGs between the experimental groups were identified using DESeq2 software. GO and KEGG analyses were performed using the DAVID platform. The PPI network was explored using the STRING analysis tool (<https://www.string-db.org/>). The PANTHER classification system (<http://pantherdb.org/>) was employed to determine the "Molecular Function" and "Pathways" associated with these genes. Furthermore, GCBI analysis (<https://www.gcbi.com.cn/>) was conducted to identify potential proteins that may interact with SGK1. For further information, original data can be found at <http://www.ncbi.nlm.nih.gov/bioproject/830843> (BioProject ID: PRJNA830843).

**Oxygen consumption rate (OCR) and extracellular acidification rate (ECAR)**

A549 and IMR-90 were harvested and resuspended in cell medium using the previously described method^17^. The cell suspension containing 4 × 10^4^ cells per well with a volume of 100 μL was seeded into Seahorse XF24 culture microplates and incubated in a biosafety hood for 1 hour. Subsequently, the plates were transferred to a 37℃ incubator with 5% CO_2_ for 4 hours. After the initial incubation, 150 µL of media was added to each well, and then cultured overnight. The Extracellular Oxygen Consumption Assay Kit (ab197243) and Glycolysis Assay Extracellular Acidification Assay Kit (ab197244) were purchased from Abcam. Oligomycin and rotenone + antimycin A were dissolved in the detection medium, and their final concentrations were 1.5 µM and 0.5 µM, respectively, and added to the sensor cartridge. For Extracellular Acidification Rate (ECAR) analysis, cell suspension with a density of 4 × 10^4^ cells per well was added to a 24-well plate. After the baseline measurement (T0), glucose (10 mM), oligomycin (1 µM), and 2-deoxy-D-glucose (2-DG; 100 mM) were added at the indicated time.

**Determination of lactate acid, pyruvate acid, nicotinamide adenine dinucleotide (NAD^+^), nicotinamide adenine dinucleotide (NADH^+^) and ATP levels in lung tissue samples and cultured cells**

Lactate Acid, Pyruvate Acid, NAD+, NADH+, and ATP Detection Kits were got from Nanjing Jiancheng Bioengineering Institute. Fresh lung tissues were collected from WT, *p16KO*, WT+ABT263, *ApoE^-/-^*, *ApoE^-/-^p16^-/-^*, and *ApoE^-/-^*+ABT263 mice, and the levels of each metabolite were determined following the manufacturer's instructions provided with each respective kit. IMR-90 and A549 cell lines were transduced with non-targeting control (NC) and p16-overexpressing adenoviral particles, and steatosis was induced by incubating the cells with sodium palmitate (1 mmol/L) and sodium oleate (1 mmol/L) for 24 hours. Subsequently, the cells were collected, and the levels of the metabolites were assessed using the respective detection kits.

**Statistical analysis**

SPSS Statistics 19.0 software was used for statistical analysis. Data are presented as the mean ± SEM fold-changes over control and were analyzed by Student’s *t*-test and one-way ANOVA. Qualitative data are presented as percentages and were analyzed using a chi-square test as indicated. A P-value < 0.05 was considered statistically significant.

**Reference**

1. Jin J, Lv X, Chen L*, et al.* Bmi-1 plays a critical role in protection from renal tubulointerstitial injury by maintaining redox balance. *Aging Cell* 2014; **13**: 797-809.

2. Gu X, Peng CY, Lin SY*, et al.* P16(INK4a) played a critical role in exacerbating acute tubular necrosis in acute kidney injury. *Am J Transl Res* 2019; **11**: 3850-3861.

3. Sharpless NE, Bardeesy N, Lee KH*, et al.* Loss of p16Ink4a with retention of p19Arf predisposes mice to tumorigenesis. *Nature* 2001; **413**: 86-91.

4. Chang J, Wang Y, Shao L*, et al.* Clearance of senescent cells by ABT263 rejuvenates aged hematopoietic stem cells in mice. *Nat Med* 2016; **22**: 78-83.

5. Xi X, Liu S, Shi H*, et al.* Serum-glucocorticoid regulated kinase 1 regulates macrophage recruitment and activation contributing to monocrotaline-induced pulmonary arterial hypertension. *Cardiovasc Toxicol* 2014; **14**: 368-378.

6. Shi D, Zhan X, Yu X*, et al.* Inhibiting CB1 receptors improves lipogenesis in an in vitro non-alcoholic fatty liver disease model. *Lipids Health Dis* 2014; **13**: 173.

7. Lu G, Yi J, Gubas A*, et al.* Suppression of autophagy during mitosis via CUL4-RING ubiquitin ligases-mediated WIPI2 polyubiquitination and proteasomal degradation. *Autophagy* 2019; **15**: 1917-1934.

8. Long X, Zhang L, Zhang Y*, et al.* Histone methyltransferase Nsd2 is required for follicular helper T cell differentiation. *J Exp Med* 2020; **217**.

9. Jin J, Tao J, Gu X*, et al.* P16 (INK4a) Deletion Ameliorated Renal Tubulointerstitial Injury in a Stress-induced Premature Senescence Model of Bmi-1 Deficiency. *Sci Rep* 2017; **7**: 7502.

10. Wang M, Xue Y, Shen L*, et al.* Inhibition of SGK1 confers vulnerability to redox dysregulation in cervical cancer. *Redox biology* 2019; **24**: 101225.

11. Pan Y, Shu G, Fu L*, et al.* EHBP1L1 Drives Immune Evasion in Renal Cell Carcinoma through Binding and Stabilizing JAK1. *Adv Sci (Weinh)* 2023: e2206792.

12. Henrichs S, Wang B, Fukao Y*, et al.* Regulation of ABCB1/PGP1-catalysed auxin transport by linker phosphorylation. *EMBO J* 2012; **31**: 2965-2980.

13. Schafer F, Seip N, Maertens B, Block H, Kubicek J. Purification of GST-Tagged Proteins. *Methods Enzymol* 2015; **559**: 127-139.

14. Zhou X, Li Y, Wang W*, et al.* Regulation of Hippo/YAP signaling and Esophageal Squamous Carcinoma progression by an E3 ubiquitin ligase PARK2. *Theranostics* 2020; **10**: 9443-9457.

15. Wang H, Deng Y, Peng S*, et al.* RNA-Seq based transcriptome analysis in oral lichen planus. *Hereditas* 2021; **158**: 39.

16. Montero-Melendez T, Nagano A, Chelala C, Filer A, Buckley CD, Perretti M. Therapeutic senescence via GPCR activation in synovial fibroblasts facilitates resolution of arthritis. *Nat Commun* 2020; **11**: 745.

17. Zhou J, Hou C, Chen H*, et al.* P16 (I NK 4a) Deletion Ameliorates Damage of Intestinal Epithelial Barrier and Microbial Dysbiosis in a Stress-Induced Premature Senescence Model of Bmi-1 Deficiency. *Frontiers in cell and developmental biology* 2021; **9**: 671564.

**Supplemental Figures and Legends**

**Figure S1. HFD or steatosis induces p16 accumulation and cell senescence in epithelial cells and fibroblasts in the lungs of aging WT- or *ApoE*^-/-^- mice or IMR-90 and A549 cells.** Primary fibroblasts and type II alveolar epithelial cells were isolated from the lung tissue of 12-month-old WT and *ApoE^-/-^* mice on HFD or normal diet for 6 months, and cultured. (A-B) RT-qPCR analysis of *p21*, *p19* and *p53* mRNA levels in primary fibroblasts and type II alveolar epithelial cells. Values were calculated relative to the *GAPDH* mRNA loading control. n=8 biological replicates per experiment. Values are given as mean±SEM, ^**^p < 0.01, ^***^p < 0.001 compared with WT or *ApoE^-/-^* mice. Statistical analysis was performed using Student’s *t*-test. (C-D) IMR-90 and A549 cells were treated with P&O for 24 h to induce steatosis. Representative western blots showing β-gal and p16 protein expression levels. GAPDH was used as the loading control. (E-F) Protein bands were quantified by densitometric analysis and normalized to GAPDH expression levels in C and D, respectively. n=3 biological replicates per experiment. Values are presented as the mean±SEM. ^*^p < 0.05, ^**^p < 0.01 compared with negative control (NC) group. Statistical analysis was performed using unpaired Student’s *t*-test.

**Figure S2. *P16* deletion or ABT263 treatment inhibits HFD-induced pulmonary fibrosis in aging WT or *ApoE*^-/-^ mice.** Twelve-month-old WT and *ApoE^-/-^* mice were fed a HFD or normal diet and treated with ABT263 for 6 months. Twelve-month-old *p16^-/-^* and *ApoE^-/-^p16^-/-^* mice were fed a HFD without ABT263 treatment. Lung tissue obtained from these mice was subsequently analyzed. (A-B) Representative images showing lung tissue sections stained with Sirius Red. (C-D) Percentage of terminal bronchial and alveolar areas positive for Sirius Red in A and B, respectively. n=3 mice per group. Values are given as the mean ± SEM, ^**^p < 0.01, ^***^p < 0.001 compared with WT or *ApoE^-/-^* mice; ^#^p < 0.05, ^##^p < 0.01 compared with *p16^-/-^* mice. Statistical analysis was performed using one-way ANOVA.

**Figure S3. *P16* deletion or ABT263 treatment ameliorates HFD-induced pulmonary fibrosis in aging WT or *ApoE*^-/-^ mice or P&O-induced steatosis in IMR-90 and A549 cells.** Twelve-month-old WT and *ApoE^-/-^* mice were fed a HFD or normal diet and treated with ABT263 for 6 months. Twelve-month-old *p16^-/-^* and *ApoE^-/-^p16^-/-^* mice were fed a HFD without ABT263 treatment. Lung tissue obtained from these mice was subsequently analyzed. (A-B) Representative immunohistochemical images showing α-SMA and POSTN staining in the lung tissue of 18-month-old *p16^-/-^*, WT and WT+ABT263 mice. (C) Percentage of areas staining positive for α-SMA and POSTN in A and B, respectively. (D-E) Representative immunohistochemical images showing α-SMA and POSTN staining in the lung tissue of 18-month-old *ApoE^-/-^*, *ApoE^-/-^p16^-/-^* and *ApoE^-/-^*+ABT263 mice. (F) Percentage of areas staining positive for α-SMA and POSTN in D and E, respectively. n=3 mice per group. Values are given as the mean±SEM, ^**^p < 0.01, ^***^p < 0.001 compared with WT or *ApoE^-/-^* mice; ^#^p < 0.05, ^##^p < 0.01 compared with *p16^-/-^* or *ApoE^-/-^p16^-/-^* mice. Statistical analysis was performed using one-way ANOVA. IMR-90 and A549 cells were transduced with *p16* overexpression adenovirus or vehicle adenovirus and treated with P&O for 24 h to induce non-inflammatory steatosis. (G-J) Representative immunofluorescence images showing α-SMA and POSTN staining in IMR-90 and A549 cells. (K-L) Western blot analysis of α-SMA protein expression levels in IMR-90 (K) and A549 (L) cells. GAPDH was used as the loading control. (M-N) Protein bands were quantified by densitometric analysis and normalized to GAPDH expression levels in K and L, respectively. n=3 biological replicates per experiment. Values are presented as the mean±SEM. ^**^p < 0.01, ^***^p < 0.001 compared with negative control (NC) group. Statistical analysis was performed using unpaired Student’s *t*-test.

**Figure S4. *P16* deletion or ABT263 treatment inhibits cell senescence in aging HFD-fed WT or *ApoE^-/-^* mice.** Twelve-month-old WT and *ApoE^-/-^* mice were fed a HFD or normal diet and treated with ABT263 for 6 months. Twelve-month-old *p16^-/-^* and *ApoE^-/-^p16^-/-^* mice were fed a HFD without ABT263 treatment. Lung tissue obtained from these mice was subsequently analyzed. (A-L) RT-qPCR was used to examine *CDK1*, *CDKN1C*, *CCNB1*, *CCNB2*, *p53* and *p21* mRNA levels in the lungs of 18-month-old *p16^-/-^*, WT and WT+ABT263 (A-F) and *ApoE^-/-^*, *ApoE^-/-^p16^-/-^* and *ApoE^-/-^*+ABT263 (G-L) mice. Values were calculated relative to the *GAPDH* mRNA loading control. n=15 biological replicates per experiment. Values are given as mean±SEM, ^*^p < 0.05, ^**^p < 0.01, ^***^p < 0.001. Statistical analysis was performed using one-way ANOVA.

**Figure S5. P16 overexpression aggravates cell senescence in P&O-treated IMR-90 and A549 cells, and *P16* deletion or ABT263 treatment ameliorates SASP in the lungs of aging HFD-fed *ApoE^-/-^* mice.** IMR-90 and A549 cells were transduced with *p16* overexpression adenovirus or vehicle adenovirus and treated with P&O for 24 h to induce non-inflammatory steatosis. (A-B) Representative western blots showing β-gal and p53 protein expression levels in IMR-90 (A) and A549 (B) cells. GAPDH was used as the loading control. (C-D) Protein bands were quantified by densitometric analysis and normalized to GAPDH expression levels in A and B, respectively. n=3 biological replicates per experiment. Values are given as the mean±SEM. ^***^p < 0.001 compared with negative control (NC) group. Statistical analysis was performed using unpaired Student’s *t*-test. Twelve-month-old *ApoE^-/-^* mice were fed a HFD or normal diet and treated with ABT263 for 6 months. Twelve-month-old *ApoE^-/-^p16^-/-^* mice were fed a HFD without ABT263 treatment. Lung tissue obtained from these mice was subsequently analyzed. (E-G) Representative immunohistochemical images showing IL-1β, IL-6 and TNF-α staining. (H) Percentage of areas stained positive for IL-1β, IL-6 and TNF-α. n=3 mice per group. Values are presented as the mean±SEM, ^***^p < 0.001 compared with *ApoE^-/-^* mice; ^#^p < 0.05, ^##^p < 0.01, compared with *ApoE^-/-^p16^-/-^* mice. Statistical analysis was performed using one-way ANOVA. (I-M) RT-qPCR was used to assess the mRNA expression levels of SASP genes (*IL-1β*, *IL-6*, *TNF-α*, *MMP3* and *CXCL5*) in the lung tissue of different treatment groups. Values were calculated relative to the *GAPDH* mRNA loading control. n=4 mice per group. Values are given as mean±SEM, *p<0.05, **p<0.01 compared with *ApoE^-/-^* mice. Statistical analysis was performed using one-way ANOVA.

**Figure S6. Gene expression profile analysis showing that *P16* deletion affects the inflammatory response and cell cycle in the lung.** (A) Hierarchical cluster diagram of gene expression levels in the lungs of 18-month-old *p16^-/-^* (*p16*-KO) and WT mice. (B) Scatter plot graph of differential gene expression levels after *p16* deletion. (C) MA plot of differentially expressed genes after *p16* knockout. (D-E) Functional profiling showing “Biological Processes” and “Molecular Function” of all downregulated differentially expressed genes identified by RNA-seq analysis using the Panther Classification System. (F) Heatmap showing the mRNA expression levels of inflammatory response-associated genes in the lungs of 18-month-old HFD-fed WT and *p16*^-/-^ mice. (G) Heat map showing the mRNA expression levels of cell cycle-associated genes in the lungs of 18-month-old HFD-fed WT and *p16^-/-^* mice.

**Figure S7. *P16* deletion or ABT263 treatment inhibits integrin-inflammasome signaling in the lungs of aging HFD-fed WT- or *ApoE^-/-^* mice, while p16 overexpression activates integrin-inflammasome signaling in P&O-treated A549 cells.** Twelve-month-old WT and *ApoE^-/-^* mice were fed a HFD or normal diet and treated with ABT263 for 6 months. Twelve-month-old *p16^-/-^* and *ApoE^-/-^p16^-/-^* mice were fed a HFD without ABT263 treatment. Lung tissue was obtained from these mice and subsequently analyzed. (A-E) Representative immunohistochemical images showing ITGAL, ITGAM, NLRP3, NLRC4 and caspase-1 staining in the lung tissue of 18-month-old *p16^-/-^*, WT and WT+ABT263 mice. (F) Percentage of areas positive for ITGAL, ITGAM, NLRP3, NLRC4 and caspase-1. (G-K) Representative immunohistochemical images showing ITGAL, ITGAM, NLRP3, NLRC4 and caspase-1 staining in the lung tissue of 18-month-old *ApoE^-/-^*, *ApoE^-/-^p16^-/-^* and *ApoE^-/-^*+ABT263 mice. (L) Percentage of areas positive for ITGAL, ITGAM, NLRP3, NLRC4 and caspase-1. (M-N) Representative immunohistochemical images showing ASC staining in the lung tissue of 18-month-old *p16^-/-^*, WT and WT+ABT263, and *ApoE^-/-^*, *ApoE^-/-^p16^-/-^* and *ApoE^-/-^*+ABT263 mice. (O-P) Percentage of ASC-positive areas in M and N, respectively. n=3 mice per group. (Q) Representative western blot showing ITGAL, ITGAM, NLRC4, NLRP3 and caspase-1 p10 protein expression levels in the lungs of 18-month-old *ApoE^-/-^*, *ApoE^-/-^p16^-/-^* and *ApoE^-/-^*+ABT263 mice. GAPDH was used as the loading control. (R) Protein bands were quantified by densitometric analysis and normalized to GAPDH expression levels. Values are presented as mean±SEM, ***p < 0.001 compared with *ApoE^-/-^* mice; ^#^p < 0.05 compared with *ApoE^-/-^p16^-/-^* mice. Statistical analysis was performed using one-way ANOVA. A549 cells were transduced with vehicle or *p16* overexpression adenoviruses and treated with P&O for 24 h to induce non-inflammatory steatosis. (S) Representative western blot showing ITGAL, ITGAM, NLRC4, NLRP3, ASC and caspase-1 p20 protein expression levels in A549 cells. GAPDH was used as the loading control. (T) Protein bands were quantified by densitometric analysis and normalized to GAPDH expression levels. n=3 biological replicates per experiment. Values are given as the mean±SEM. ^**^p < 0.01, ^***^p < 0.001 compared with negative control (NC) group. Statistical analysis was performed using unpaired Student’s *t*-test.

**Figure S8. *P16* deletion or ABT263 treatment inhibits integrin-inflammasome pathway-related genes in the lungs of aging HFD-fed WT- or *ApoE^-/-^* mice.** Twelve-month-old WT and *ApoE^-/-^* mice were fed a HFD or normal diet and treated with ABT263 for 6 months. Twelve-month-old *p16^-/-^* and *ApoE^-/-^p16^-/-^* mice were fed a HFD without ABT263 treatment. Lung tissue from these mice was collected and analyzed. (A-P) RT-qPCR analysis of *ITGAM*, *ITGAL*, *NLRC4*, *ITGB2L*, *ITGB2*, *TXK*, *NAIP6* and *NAIP5* mRNA levels in the lungs of 18-month-old *p16^-/-^*, WT and WT+ABT263 mice (A-C and G-K) and *ApoE^-/-^*, *ApoE^-/-^p16^-/-^* and *ApoE^-/-^*+ABT263 mice (D-F and L-P). Values were calculated relative to the *GAPDH* mRNA loading control. n=15 biological replicates per experiment. Values are given as the mean±SEM, ^*^p < 0.05, ^**^p < 0.01, ^***^p < 0.001. Statistical analysis was performed using one-way ANOVA.

**Figure S9. *P16* deletion or ABT263 treatment affects cell metabolism in the lungs of aging HFD-fed WT or *ApoE^-/-^* mice.** Twelve-month-old WT and *ApoE^-/-^* mice were fed a HFD or normal diet and treated with ABT263 for 6 months. Twelve-month-old *p16^-/-^* and *ApoE^-/-^p16^-/-^* mice were fed a HFD without ABT263 treatment. Lung tissue was collected from these mice and subsequently analyzed. (A-N) RT-qPCR was used to determine *HKDC1*, *DGKG*, *DGKB*, *FBP1*, *FBP2*, *PKLR* and *GCK* mRNA expression levels in the lungs of 18-month-old *p16^-/-^*, WT and WT+ABT263 mice (A-G) and *ApoE^-/-^*, *ApoE^-/-^p16^-/-^* and *ApoE^-/-^*+ABT263 mice (H-N). Values were calculated relative to the *GAPDH* mRNA loading control. n=15 biological replicates per experiment. Values are given as the mean±SEM, ^*^p < 0.05, ^**^p < 0.01, ^***^p < 0.001 compared with WT or *ApoE^-/-^* mice. Statistical analysis was performed using one-way ANOVA.

**Figure S10. *P16* deletion or clearance of senescent cells decreases glycolysis in the lungs of aging HFD-fed WT or *ApoE^-/-^* mice.** Twelve-month-old WT and *ApoE^-/-^* mice were fed a HFD or normal diet and treated with ABT263 for 6 months. Twelve-month-old *p16^-/-^* and *ApoE^-/-^p16^-/-^* mice were fed a HFD without ABT263 treatment. Lung tissue was collected from these mice and subsequently analyzed. (A-F) NAD^+^ levels, NADH levels and the NAD^+^/NADH ratio were detected in the lungs of 18-month-old *p16^-/-^*, WT and WT+ABT263 mice (A-C) and *ApoE^-/-^*, *ApoE^-/-^p16^-/-^* and *ApoE^-/-^*+ABT263 mice (D-F). n=4 biological replicates per experiment. Values are given as the mean±SEM, ^*^p < 0.05, ^**^p < 0.01, ^***^p < 0.001 compared with WT or *ApoE^-/-^* mice. Statistical analysis was performed using one-way ANOVA.

**Figure S11. HFD promotes activation of the integrin-inflammasome pathway in physiologically aged mice.** The mRNA expression levels of integrin-inflammasome pathway-associated genes were examined in the lungs of 18-month-old WT mice fed a HFD or normal diet. (A-D) RT-qPCR was used to detect *CXCL5*, *IL-6*, *MMP3*, *TNF-α*, *IL-1β*, *CDK1*, *CCNB2*, *CDKN1C*, *CCNB1*, *ITGAM*, *ITGAL*, *NLRC4*, *ITGB2L*, *ITGB2*, *TXK*, *NAIP6* and *NAIP5* mRNA expression levels in the lung tissue. Values were calculated relative to the *GAPDH* mRNA loading control. n=8 biological replicates per experiment. Values are given as the mean±SEM, ^**^p < 0.01, ***p < 0.001 compared with normal diet-fed mice. Statistical analysis was performed using unpaired Student’s *t*-test. (E) Representative western blot showing ITGAL, ITGAM, NLRC4, NLRP3, ASC, caspase 1 p10, cleaved-IL-1β, IL-6 and TNF-α protein expression levels. GAPDH was used as the loading control. (F) Protein bands were quantified by densitometric analysis and normalized to GAPDH expression levels. n=3 biological replicates per experiment. Values are given as the mean±SEM. ^*^p < 0.05, ^**^p < 0.01, ^***^p < 0.001 compared with normal diet-fed mice. Statistical analysis was performed using unpaired Student’s *t*-test.

**Figure S12. P16 interacts with SGK1 and inhibits K48-linked polyubiquitin-dependent degradation of SGK1.** Steatosis was induced in IMR-90 and A549 cell lines by treatment with P&O. (A) Representative immunofluorescence images showing the co-localization of p16 and SGK1 in A549 cells. (B-C) IMR-90 cells were transduced with Flag-p16 overexpression adenovirus and treated with P&O for 24 h. Cells were collected and analyzed by western blotting. (B) Representative western blot showing SGK1 protein expression levels in IMR-90 cells. (C) Quantification of SGK1 protein expression levels. Values are expressed relative to GAPDH loading control. (D) GST-pull down experiments were performed by incubating purified GST-P16 and GST protein with A549 cell lysates for 4 days. The assay demonstrated an *in vitro* interaction between p16 and SGK1 in the cell lysate of A549 cells. (E-F) IMR-90 and A549 cells were transduced with Flag-p16 overexpression adenovirus and treated with P&O for 24 h. Cycloheximide (CHX; 100 μg/ml) was added to the cells at the indicated time points, and the half-life of SGK1 was examined by western blotting in IMR-90 (E) and A549 (F) cells. (G-K) IMR-90 and A549 cells were treated with or without P&O. Co-IP was performed on the cell lysate using an antibody against SGK1. Western blot analysis was used to determine the levels of SGK1 ubiquitination, K48-linked ubiquitination and K63-linked ubiquitination. (G) *In vivo* ubiquitination assay showing the levels of SGK1 ubiquitination in A549 cells. (H-I) *In vivo* ubiquitination assay showing K63-linked ubiquitination levels of SGK1 in IMR-90 and A549 cells. (J-K) *In vivo* ubiquitination assay showing the K48-linked ubiquitination levels of SGK1 in IMR-90 and A549 cells. (L) P&O-treated A549 cell lines were transduced with Flag-p16 or Flag-NC overexpression adenovirus. Co-IP was carried out on the cell lysate using an antibody against SGK1. Western blot analysis was used to determine the level of interaction between SGK1 and NEDD4L in A549 cells.

**Figure S13 The SGK1 specific inhibitor EMD638683 ameliorates HFD induced- pulmonary senescence, inflammasome activation, and SASP.** Twelve-month-old male WT mice were fed a HFD, and administered EMD638383 i.p. daily (20 mg/kg body weight) for 6 months. Lung tissue was collected and analyzed. Representative immunohistochemical images and statistical analysis of positive-stained areas for p19, IL-1β, IL-6, TNF-α, ITGAL, NLRC4, NLRP3, ASC and caspase-1 in the lungs of 18-month-old WT and WT+EMD638683 mice. n=3 biological replicates per experiment. Values are given as the mean±SEM. ^***^p < 0.001 compared with WT mice. Statistical analysis was performed using unpaired Student’s *t*-test.

**Figure S14. EMD638683 inhibits p16-mediated activation of the integrin-inflammasome pathway in P&O-treated IMR-90 cells.** IMR-90 cells were transduced with vehicle or *p16* overexpression adenoviruses and treated with P&O to induce non-inflammatory steatosis. Cells were also treated with EMD638683 (10 μM) for 24 h. (A) Representative western blot showing ASC, caspase-1 p10, cleaved-IL-1β, IL-6, ITGAL, ITGAM, NLRC4 and NLRP3 protein expression levels in IMR-90 cells. GAPDH was used as the loading control. (B) Protein bands were quantified by densitometric analysis and normalized to GAPDH expression levels. n=3 biological replicates per experiment. Values are given as the mean±SEM. ^*^p < 0.05, ^**^p < 0.01, ^***^p < 0.001 compared with NC group; ^#^*p* < 0.05, ^##^*p* < 0.01, ^###^*p* < 0.001 compared with *p16*-OE group. Statistical analysis was performed using one-way ANOVA.

**Figure S15. SGK1 siRNA inhibits p16-mediated activation of the integrin-inflammasome pathway in P&O-treated IMR-90 and A549 cell lines.** the IMR-90 and A549cell lines were induced with *p16* over-expression adenovirus and transfected with small interfering RNA (si-SGK1) targeting to SGK1 *in vitro*. After treating with P&O for 24h, cell lysates were collected and for further immunoblotting detection. Representative western blot showing SGK1, ASC, caspase-1 p10, cleaved-IL-1β, IL-6, ITGAL, ITGAM, NLRC4 and NLRP3 protein expression levels in (A) IMR-90 cells and (C) A549 cells. GAPDH was used as the loading control. (B and D) Protein bands were quantified by densitometric analysis and normalized to GAPDH expression levels. n=3 biological replicates per experiment. Values are given as the mean±SEM. ^*^p < 0.05, ^**^p < 0.01, ^***^p < 0.001 compared with NC group; ^#^*p* < 0.05, ^##^*p* < 0.01, ^###^*p* < 0.001 compared with *p16*-OE group. Statistical analysis was performed using one-way ANOVA.

**Table S1-S5**

**Table S1** Chow composition of high fat diet and normal diet

| Normal diet | gm% | Kcal% |
| --- | --- | --- |
| Protein | 18.8 | 20.54 |
| Fat | 12.79 | 12.79 |
| Carbohydrate | 66.67 | 66.67 |

| High fat diet | gm% | Kcal% |
| --- | --- | --- |
| Protein | 26.2 | 20 |
| Fat | 34.9 | 60 |
| Carbohydrate | 26.3 | 20 |

**Table S2 Antibodies and reagents used in Western blots or Co-IP experiments**

| **Name** | **Catalog Number** | **Company** | **Dilution** |
| --- | --- | --- | --- |
| Beta Galactosidase | 15518-1-AP | Proteintech | 1:500 |
| p16^INK4a^ (p16) | ab108349 | Abcam | WB 1:1000  IP 1:50 |
| p16^INK4a^ (p16) | ab211542 | Abcam | WB 1:1000  IP 1:50 |
| Periostin (POSTN) | ab152099 | Abcam | 1:500 |
| Collagen I | GTX26308 | Gene Tex | 1:1000 |
| α-SMA | 14395-1-AP | Proteintech | 1:2000 |
| p53 | 2524 | Cell Signaling Technology | 1:1000 |
| p19 | 10272-2-AP | Proteintech | 1:1000 |
| IL-6 | GTX110527 | Gene Tex | 1:1000 |
| TNF-α | ab183218 | Abcam | 1:1000 |
| IL-1β | ab254360 | Abcam | 1:1000 |
| p-p65(S536) | 3033 | Cell Signaling Technology | 1:1000 |
| NLRP3 | 15101 | Cell Signaling Technology | 1:1000 |
| NLRC4 | ab201792 | Abcam | 1:1000 |
| Caspase-1 | 83383 | Cell Signaling Technology | 1:500 |
| ASC/TMS1 | GTX55818 | Gene Tex | 1:1000 |
| Integrin Subunit Alpha M (ITGAM) | ab133357 | Abcam | 1:1000 |
| Integrin Subunit Alpha L (ITGAL) | DF6525 | Affinity | 1:1000 |
| SGK1 | ab32374 | Abcam | 1:500 |
| Ubiquitin | 3936 | Cell Signaling Technology | 1:2000 |
| K48-linkage Specific Polyubiquitin | 8081 | Cell Signaling Technology | 1:1000 |
| K63-linkage Specific Polyubiquitin | 5621 | Cell Signaling Technology | 1:1000 |
| NEDD4L | 4013 | Cell Signaling Technology | 1:1000 |
| DYKDDDDK Tag | 8146 | Cell Signaling Technology | WB 1:3000  IP 1:100 |
| DYKDDDDK Tag | 20543-1-AP | Proteintech | WB 1:5000  IP 1:50 |
| HA Tag | 51064-2-AP | Proteintech | WB 1:1000  IP 1:50 |
| HA Tag | 65738 | Cell Signaling Technology | WB 1:1000  IP 1:50 |
| Myc Tag | 2278 | Cell Signaling Technology | WB 1:1000  IP 1:100 |
| Myc Tag | 2276 | Cell Signaling Technology | WB 1:1000  IP 1:100 |
| HRP-conjugated Affinipure Goat Anti-Rabbit IgG(H+L) | SA00001-2 | Proteintech | 1:5000 |
| HRP-conjugated Affinipure Goat Anti-Mouse IgG(H+L) | SA00001-1 | Proteintech | 1:5000 |
| SuperSignal™ West Pico PLUS | 34577 | Thermo Fisher Scientific | According to  instructions |

**Table S3 Antibodies used in immunohistochemical staining**

| **Name** | **Catalog Number** | **Company** | **Dilution** |
| --- | --- | --- | --- |
| Beta Galactosidase | 15518-1-AP | Proteintech | 1:300 |
| p16^INK4a^ (p16) | ab108349 | Abcam | 1:500 |
| Periostin (POSTN) | ab152099 | Abcam | 1:500 |
| Collagen I | GTX26308 | Gene Tex | 1:250 |
| α-SMA | 14395-1-AP | Proteintech | 1:800 |
| p53 | 2524 | Cell Signaling Technology | 1:300 |
| p19 | 10272-2-AP | Proteintech | 1:300 |
| IL-6 | GTX110527 | Gene Tex | 1:800 |
| TNF-α | ab183218 | Abcam | 1:800 |
| IL-1β | ab254360 | Abcam | 1:500 |
| NLRP3 | 15101 | Cell Signaling Technology | 1:200 |
| NLRC4 | ab201792 | Abcam | 1:200 |
| Caspase-1 | 83383 | Cell Signaling Technology | 1:300 |
| ASC/TMS1 | GTX55818 | Gene Tex | 1:300 |
| Integrin Subunit Alpha M(ITGAM) | ab133357 | Abcam | 1:300 |
| Integrin Subunit Alpha L (ITGAL) | DF6525 | Affinity | 1:300 |

**Table S4 Antibodies used in immunofluorescence stainings**

| **Name** | **Catalog Number** | **Company** | **Dilution** |
| --- | --- | --- | --- |
| Beta Galactosidase | sc-377257 | Santa Cruz | 1:300 |
| p16^INK4a^(p16) | 10883-1-AP | Proteintech Group | 1:200 |
| Surfactant protein c | sc-518029 | Santa Cruz | 1:300 |
| α-SMA | MAB12016 | Abnova | 1:300 |
| Myc Tag | 2276 | Cell Signaling Technology | 1:600 |

**Table S5 Primers for qPCR**

| **Name** | **S/AS** | **Sequence** |
| --- | --- | --- |
| *CDKN1C* | S  AS | 5′- CGAGGAGCAGGACGAGAATC -3′  5′-GAAGAAGTCGTTCGCATTGGC-3 |
| *CCNB2* | S  AS | 5′- GCCAAGAGCCATGTGACTATC -3′  5′-CAGAGCTGGTACTTTGGTGTTC-3 |
| *CCNB1* | S  AS | 5′-AAGGTGCCTGTGTGTGAACC -3′  5′-GTCAGCCCCATCATCTGCG-3 |
| *CDK1* | S  AS | 5′-AGAAGGTACTTACGGTGTGGT -3′  5′-GAGAGATTTCCCGAATTGCAGT-3 |
| *CXCL5* | S  AS | 5′-TCCAGCTCGCCATTCATGC-3′  5′-TTGCGGCTATGACTGAGGAAG-3 |
| *DGKG* | S  AS | 5′-GTATCAAGTGCTACCAGAGTGTC-3′  5′-CTGTCGATAATTCACATTTGCGG-3 |
| *DGKB* | S  AS | 5′-ATGAAGACCTTTCTGGAAGCTG-3′  5′-TTTACATTTGGGCTAGAATGGGG-3 |
| *FBP1* | S  AS | 5′-CACCGCGATCAAAGCCATCT-3′  5′-AGGTAGCGTAGGACGACTTCA-3′ |
| *FBP2* | S  AS | 5′-ACCCTGACCCGTTACGTTATG-3′  5′-ACATTCACGCTCCCCGAAATC-3′ |
| *GCK* | S  AS | 5′-TGAGCCGGATGCAGAAGGA-3′  5′-GCAACATCTTTACACTGGCCT-3′ |
| *HKDC1* | S  AS | 5′-ATGTTTGCAGTACACTTGGTGG-3′  5′-AGGGTCTCATCCGAGAGCC-3′ |
| *ITGAM* | S  AS | 5′-ATGGACGCTGATGGCAATACC-3′  5′-TCCCCATTCACGTCTCCCA-3′ |
| *ITGAL* | S  AS | 5′-CCAGACTTTTGCTACTGGGAC-3′  5′-GCTTGTTCGGCAGTGATAGAG-3′ |
| *ITGB2* | S  AS | 5′-CAGGAATGCACCAAGTACAAAGT-3′  5′-CCTGGTCCAGTGAAGTTCAGC-3′ |
| *ITGB2L* | S  AS | 5′-CACTGTCTCAGTTGTGTACCAAG-3′  5′-GCTCTGGTGTATCACAGCGAA-3′ |
| *IL-1β* | S  AS | 5′-GCAACTGTTCCTGAACTCAACT-3′  5′-ATCTTTTGGGGTCCGTCAACT-3′ |
| *IL-6* | S  AS | 5’-GTTGCCTTCTTGGGACTGATG-3’  5’-ATCAGAATTGCCATTGCACAA-3’ |
| *MMP3* | S  AS | 5′-ACATGGAGACTTTGTCCCTTTTG-3′  5′-TTGGCTGAGTGGTAGAGTCCC-3′ |
| *NLRC4* | S  AS | 5′-ATCGTCATCACCGTGTGGAG-3′  5′-GCCAGACTCGCCTTCAATCA-3′ |
| *NAIP5* | S  AS | 5′-TGCCAAACCTACAAGAGCTGA-3′  5′-CAAGCGTTTAGACTGGGGATG-3′ |
| *NAIP6* | S  AS | 5′-TACAGGGAGTTTACAAGACCCC-3′  5′-AGTGGCCTGGAGAGACTCAG-3′ |
| *PKLR* | S  AS | 5′-TCAAGGCAGGGATGAACATTG-3′  5′-CACGGGTCTGTAGCTGAGTG-3′ |
| *TXK* | S  AS | 5′-ACAGGTGAGAACTCAGATAAGCC-3′  5′-GGCAGGAAGTCATAAAGAGCCT-3′ |
| *TNF-α* | S  AS | 5’-TCCCTCTCATCAGTTCTATGG-3’  5’-ACTTGGTGGTTTGCTACGAC-3’ |
| *GAPDH* | S  AS | 5’-CATTTCACTCAAGGTTGTCAGC-3’  5’-ATCATACTTGGCAGGTTTCTCC-3’ |

S, sense; AS, antisense

**Table S6 siRNAs against Human SGK1**

| **Name** | **S/AS** | **Sequence** |
| --- | --- | --- |
| siSGK1#1 | S  AS | 5′-GCCAAUAACUCCUAUGCAUTT-3′  5′-AUGCAUAGGAGUUAUUGGCTT-3′ |
| siSGK1#2 | S  AS | 5′-CCGCCAGCUGACAGGACAUTT-3′  5′-AUGUCCUGUCAGCUGGCGGTT-3′ |
