# Supplementary figures and images for: Correction to “Inflammasome activation and metabolic remodeling in p16‐positive aging cells aggravates high‐fat diet‐induced lung fibrosis by inhibiting NEDD4L‐mediated K48‐polyubiquitin‐dependent degradation of SGK1”
Source: Clin Transl Med. 2025 Jul 24;15(7):e70379. doi: 10.1002/ctm2.70379 (PMC12290014; doi:10.1002/ctm2.70379)

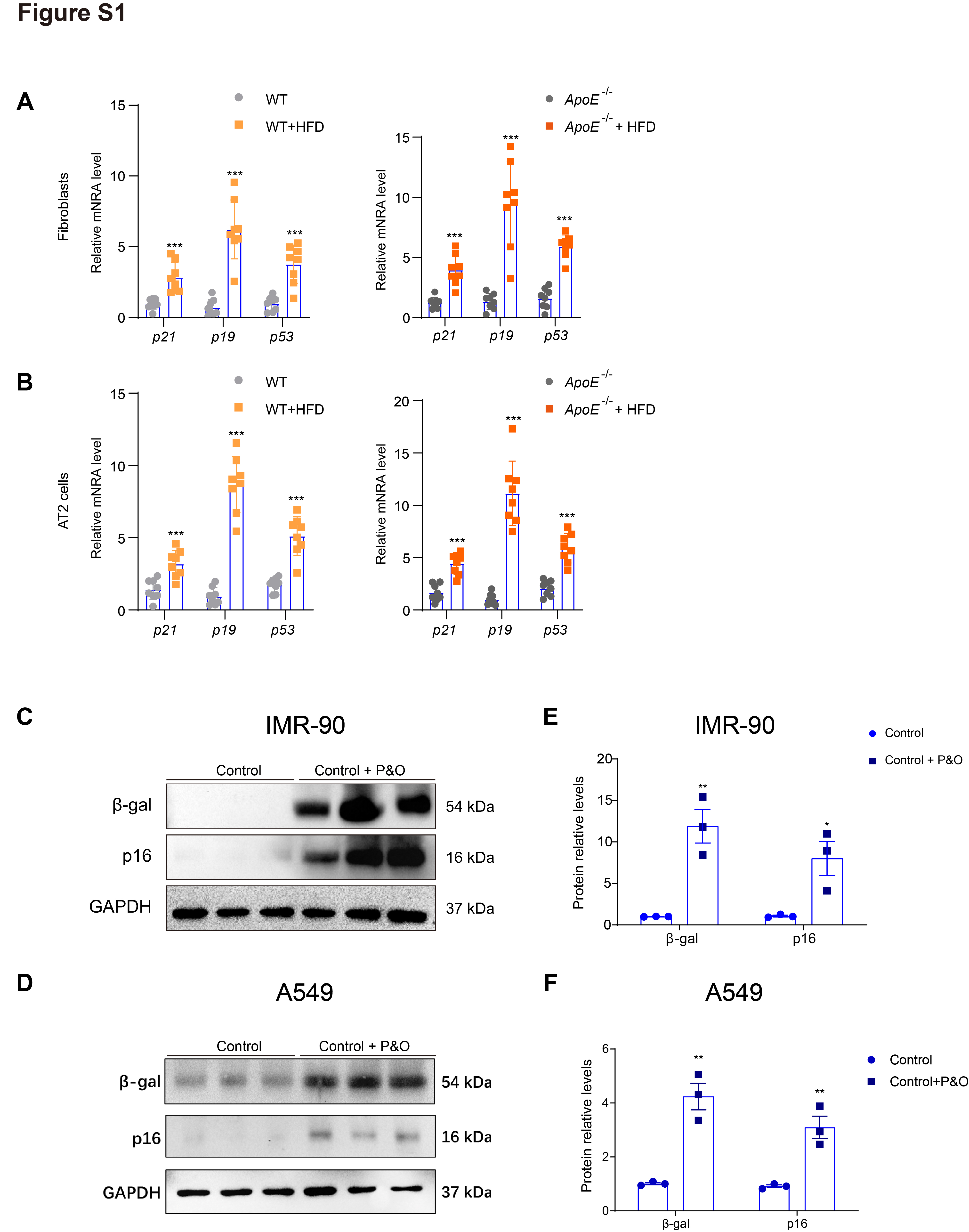

Supplement: Supplementary file 1 — Supporting infomation [file CTM2-15-e70379-s002.jpg]

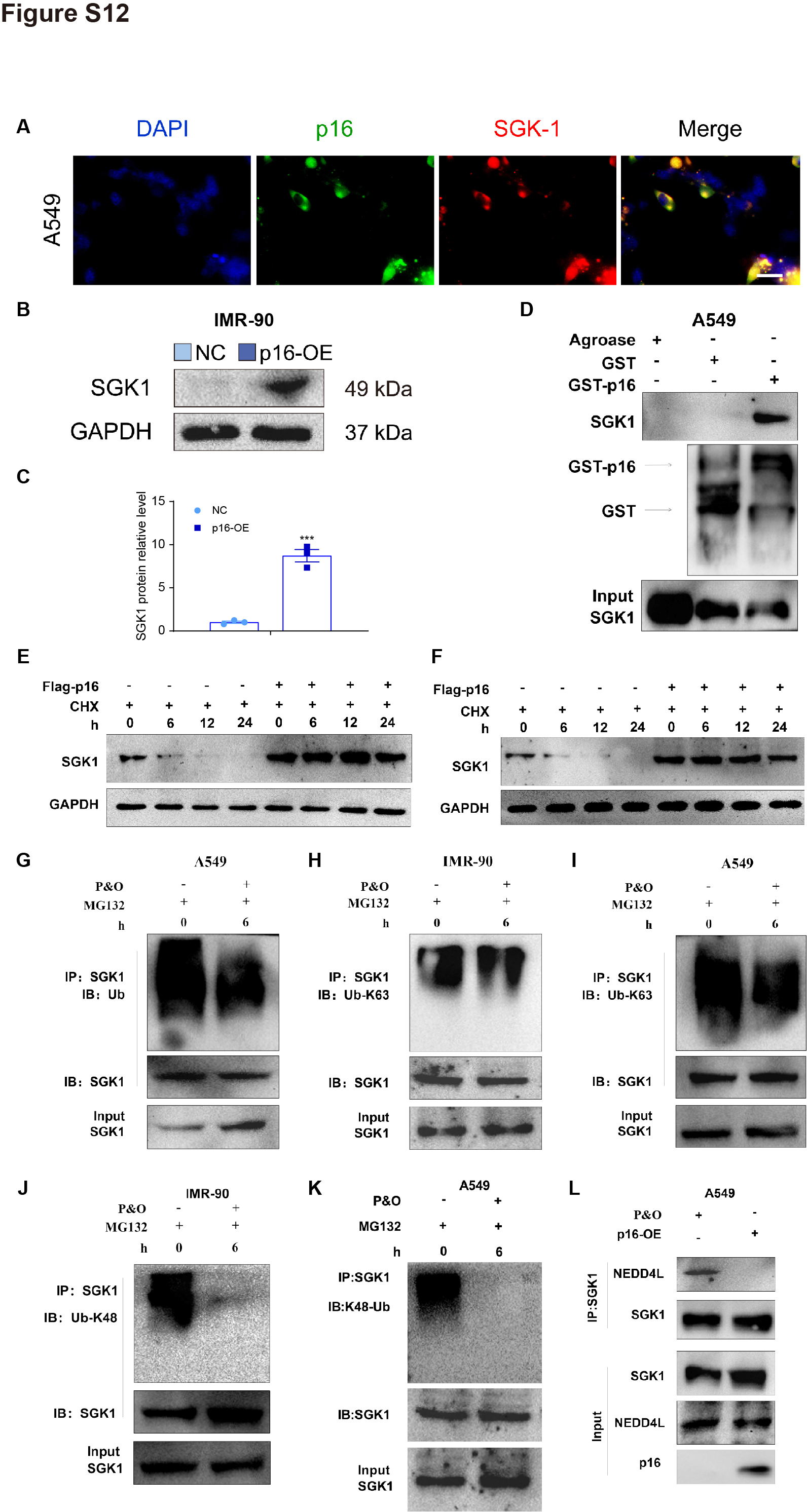

Supplement: Supplementary file 2 — Supporting infomation [file CTM2-15-e70379-s001.jpg]
